# Supplementary material for: A highly accurate risk factor-based XGBoost multiethnic model for identifying patients with skin cancer
Source: Nat Commun. 2025 Oct 29;16:9542. doi: 10.1038/s41467-025-64556-y (PMC12572141; doi:10.1038/s41467-025-64556-y)
Supplement: Supplementary file 1 — Supplementary Information [file 41467_2025_64556_MOESM1_ESM.pdf]

SUPPLEMENTARY FIGURES

Figure S1: Differences in age at diagnosis across genetic ancestries

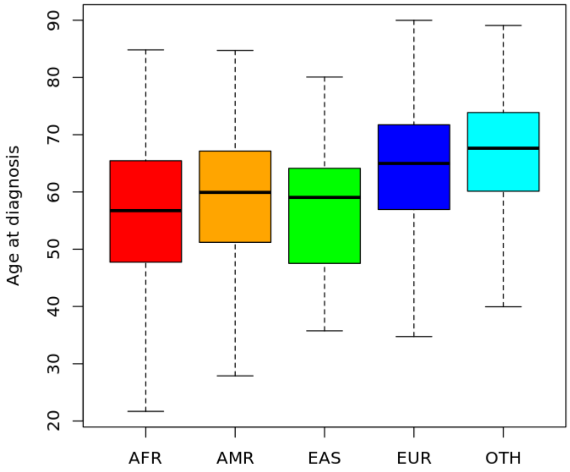

Boxplots showing the differences in age at diagnosis across populations. AFR, AMR and EAS individuals tend to be diagnosed for skin cancer at an earlier age than EUR individuals (AFR 8.3 years earlier, p = 1e-10; AMR 5.08 years earlier, p = 7e-9; and EAS 6.0 years earlier, p = 0.004, log-rank test). The central line within each box represents the median, the box edges indicate the 25th and 75th percentiles (interquartile range, IQR), and the whiskers extend to the most extreme data points within 1.5 × IQR from the quartiles.

Figure S2: Differences in incidence and age at diagnosis in genetically admixed individuals

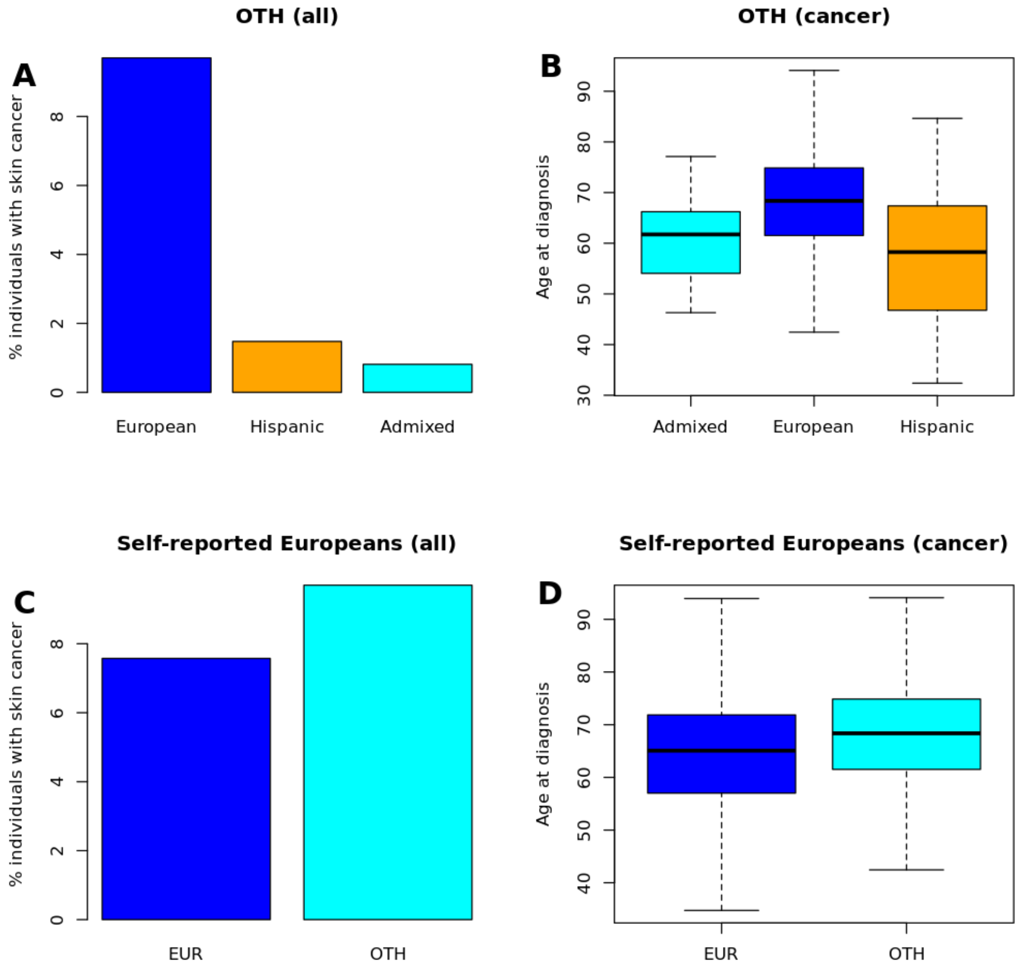

Barplots and boxplots plots showing for any type of skin cancer: A) All OTH individuals (healthy and skin cancer patients) grouped according to their self-reported ancestry (European, Hispanic or Latino, and Admixed); B) only OTH skin cancer patients grouped according to their self-reported ancestry (Hispanic or Latino, European and Admixed); C) All individuals (healthy and skin cancer patients) who self-report as European grouped according to their genetic ancestry (OTH and EUR); D) only skin cancer patients who self-report as European grouped according to their genetic ancestry (OTH and EUR). Only populations with at least 20 skin cancer patients are shown. OTH individuals who self-reported as European have a higher incidence and later age at diagnosis compared with other OTH individuals who self-reported as Admixed (multiple ancestries reported) or Hispanic (p = 5.0e-20 and p = 3.9e-17, respectively, log-rank test). Comparison between OTH individuals who self-report as European and individuals of genetic EUR ancestry, and found that, while they do not display significant differences in incidence (p = 0.82, log-rank test), they are diagnosed at a significantly later age (p = 1.2e-15, log-rank test). In the boxplots, the central line within each box represents the median, the box edges indicate the 25th and 75th percentiles (interquartile range, IQR), and the whiskers extend to the most extreme data points within 1.5 × IQR from the quartiles.

Figure S3: differences in genotype PC values between all individuals and skin cancer patients by population

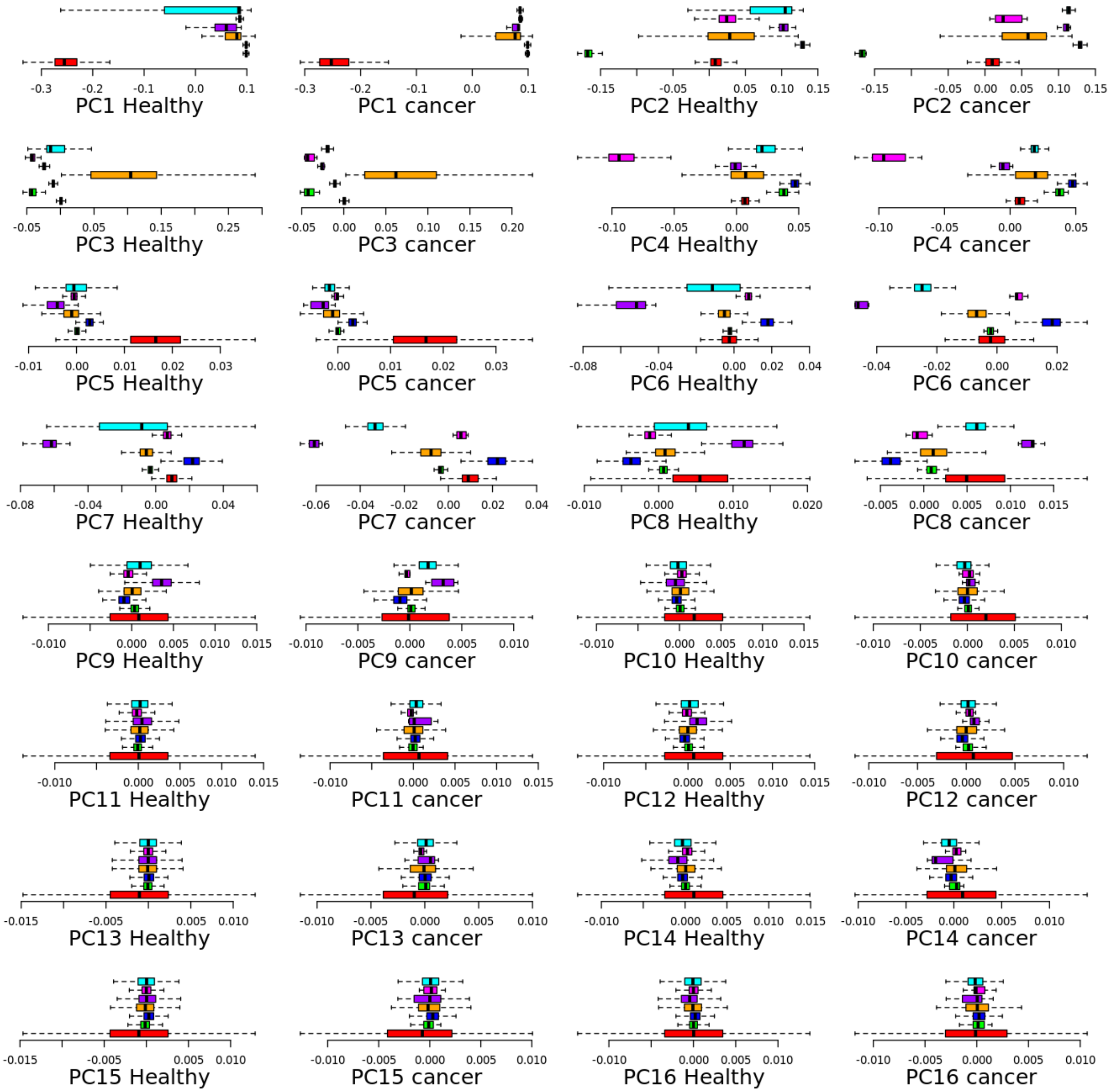

Boxplots showing the associations between each genetic ancestry and genotype PCs. For each genotype PC two plots are shown: healthy individuals (i.e. AoU participants that were not diagnosed with skin cancer) and skin cancer patients. Population colors are as in Figure 2: red = AFR; green = EAS; blue = EUR; orange = AMR; aquamarine = MID; purple = SAS; magenta = Pacific Islanders; cyan = OTH. The central line within each box represents the median, the box edges indicate the 25th and 75th percentiles (interquartile range, IQR), and the whiskers extend to the most extreme data points within 1.5 × IQR from the quartiles.

Figure S4: Wealth influences skin cancer outcomes in European individuals

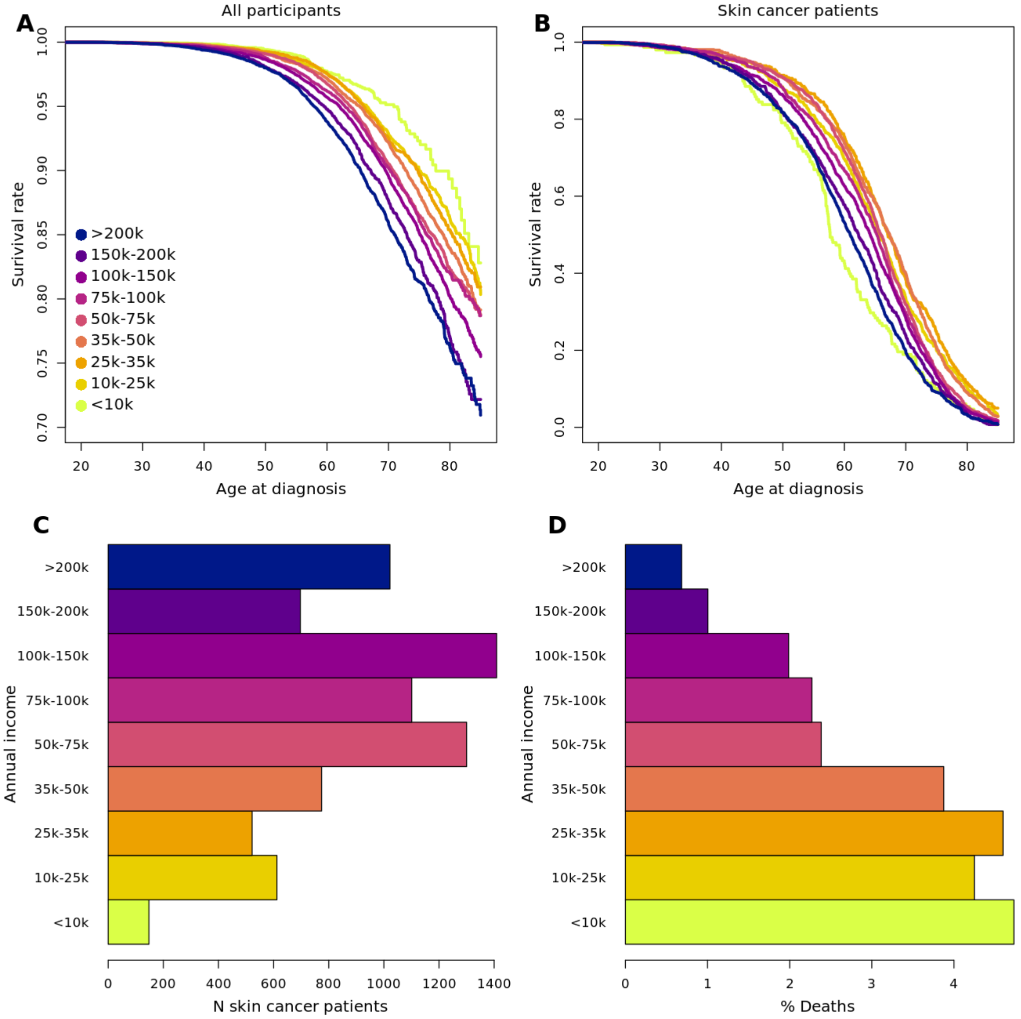

A-B) Survival plots showing by annual income: A) All EUR individuals grouped according to their annual income; B) only EUR skin cancer patients grouped according to their annual income. The plots show that, independent of their EUR ancestry, wealthier individuals tend to be diagnosed at a higher rate and earlier. In panel A, X axis indicates age at diagnosis for skin cancer patients, age at last follow-up for all other individuals.

C) Barplot showing the number of AoU EUR individuals with skin cancer in each annual income category.

D) Barplot showing the percentage of EUR individuals with skin cancer that died over the total number of individuals diagnosed with skin cancer in each annual income class. The plot shows that lower-income individuals diagnosed with skin cancer are more likely to die than wealthier individuals.

Figure S5: Stratification of individuals with skin cancer using logistic regression

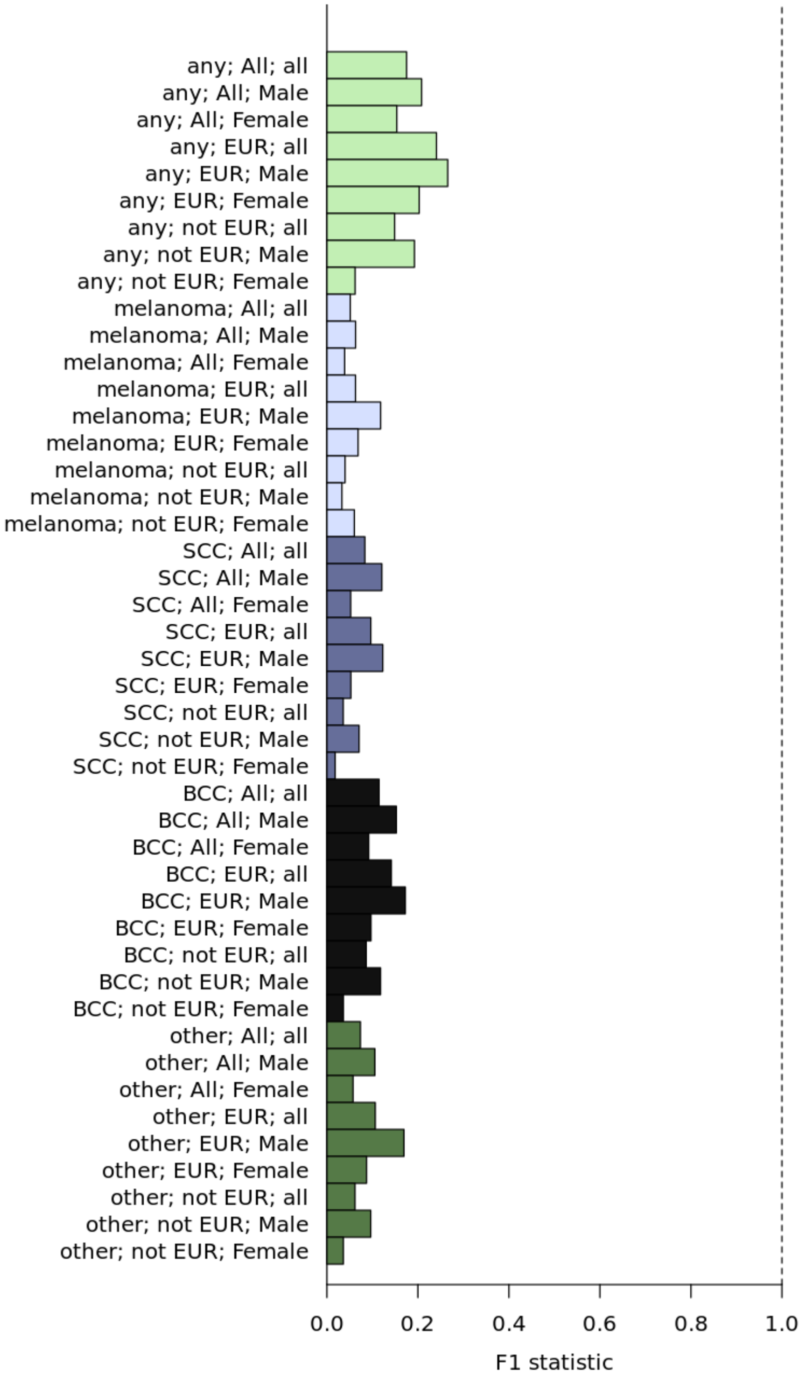

A) Barplot showing the F1 statistic of predictions using logistic regression for 45 models which consist of combinations of skin cancer categories (any, melanoma, SCC, BCC, other), populations (Europeans: EUR, non-Europeans: not EUR, All – both EUR and not EUR), sexes (Male, Female, all – both Males and Females). F1 statistics were calculated on the cross-validated training set (see Methods).

Figure S6: Stratification of individuals with skin cancer removing one variable at a time from training set

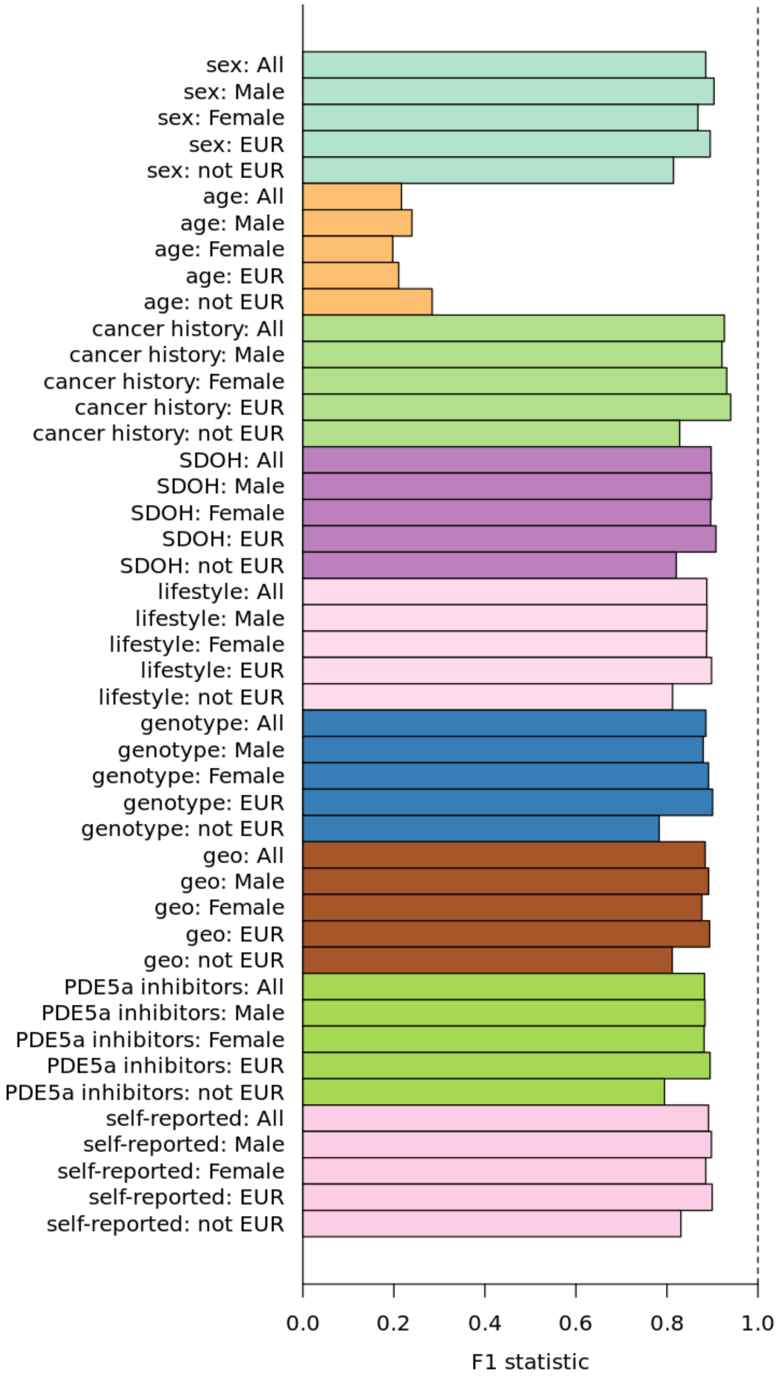

Barplots showing the F1 statistic on the cross-validation training set from eight models trained after removing one variable (sex, age, cancer history, SDOH, lifestyle, genotype PCs, geo=longitude and latitude, PDE5A inhibitors, self-reported ancestry) associated with skin cancer at a time (Table S6). SDOH include: annual income, education level, health insurance, living situation, latitude, and longitude; lifestyle includes daily and yearly alcohol intake and smoking. Each color represents the removal of a single variable from the general model (any skin cancer, all participants and both sexes) during training.

The F1 statistic results are shown when considering All validation participants, Males only, Females only, Europeans only, and non-Europeans only.

Figure S7: SHAP plots showing the influence of each variable on skin cancer prediction

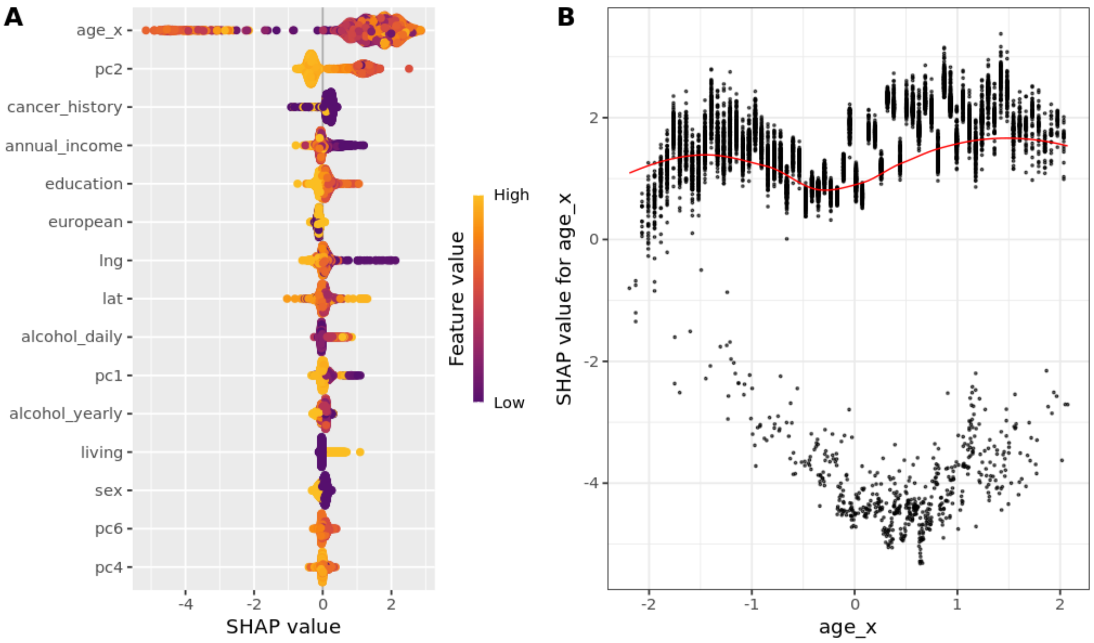

A) SHAP summary plot showing feature importance. Each dot represents a SHAP value for an individual observation. Features are ranked by mean absolute SHAP value (i.e., importance). The X-axis shows the impact of each feature on the model's prediction. Color indicates the raw value of the feature (purple = low, yellow = high). Features with greater spread and higher magnitude contribute more strongly to predictions. The plot was generated using the *sv_importance* function in shapviz 0.9.7 in R.

B) SHAP dependence plot for the feature "age". The X-axis shows the raw values of the "age" feature, and the Y-axis shows the SHAP values (i.e., the feature's contribution to the prediction). Each point represents one observation. The plot shows how "age" influences predictions across its range in a non-linear fashion. The plot was generated with the *shap.plot.dependence* function in SHAPforxgboost 0.1.3.

Supplementary Tables

Table S1: Correspondence between self-reported and PCA-based ancestry

The table shows the differences between self-reported race and ethnicity (columns) and genetic ancestry (rows), as defined by the genotype PCA.

Ancestry	More than one population reported (Admixed)	African	Asian	European	Hispanic	Middle Eastern	Pacific	Total
AFR	686	48189	<100	<100	1802	<100	<100	50765
AMR	<100	<100	<100	660	38304	<100	<100	39047
EAS	<100	<100	5057	<100	<100	<100	<100	5225
EUR	649	<100	<100	115104	990	<100	<100	116847
MID	<100	<100	<100	<100	<100	434	<100	505
OTH	2213	874	202	8125	5549	732	<100	17765
SAS	<100	<100	2024	<100	<100	<100	<100	2128
No WGS	2941	26513	6314	94838	26288	1040	171	158105
TOTAL (WGS)	3667	49141	7319	123961	46689	1287	218	232282
TOTAL	6608	75654	13633	218799	72977	2327	389	390387

Table S2: Associations between each population and skin cancer risk

For each combination of skin cancer category (SCC, melanoma, BCC, any, other) and population (AFR, AMR, EAS, EUR, MID, OTH, SAS) shown are enrichments in terms of odds ratios, 95% CI, two-sided p-value (Fisher's exact test) and Benjamini-Hochberg's adjusted p-value used to examine differences in risk of skin cancer between populations. We also show median age at diagnosis, median age at diagnosis of other population (all other populations), p-value and Benjamini-Hochberg's adjusted p-value (log-rank test) used to examine differences in age of diagnosis between populations.

Cancer	Population	Participants	Odds ratio	95% CI	p-value (Fisher)	adjusted p-value (Fisher)	Median age at diagnosis	median age at diagnosis (other populations)	p-value (log-rank)	adjusted p-value (log-rank)
SCC	AFR	50,765	0.070	0.053-0.091	2.92E-230	1.06E-229	57.851	67.556	1.53E-10	2.04E-09
melanoma	AFR	50,765	0.046	0.03-0.067	6.67E-174	2.22E-173	61.318	62.532	8.74E-01	1.00E+00
BCC	AFR	50,765	0.009	0.005-0.014	0.00E+00	0.00E+00	66.437	65.684	7.48E-01	9.65E-01
any	AFR	50,765	0.051	0.043-0.06	0.00E+00	0.00E+00	56.734	65.160	4.44E-11	8.87E-10
other	AFR	50,765	0.073	0.056-0.093	5.53E-253	2.21E-252	51.934	61.896	2.90E-10	2.90E-09
BCC	AMR	39,047	0.099	0.081-0.119	1.70E-296	8.52E-296	62.833	65.730	5.07E-02	1.11E-01
other	AMR	39,047	0.141	0.114-0.174	5.52E-148	1.58E-147	57.789	61.753	5.25E-02	1.11E-01
melanoma	AMR	39,047	0.114	0.083-0.152	3.85E-101	1.03E-100	56.058	62.668	3.78E-04	1.26E-03
any	AMR	39,047	0.131	0.116-0.148	0.00E+00	0.00E+00	59.958	65.184	1.49E-09	1.19E-08
SCC	AMR	39,047	0.107	0.082-0.137	4.12E-151	1.27E-150	61.568	67.551	5.11E-04	1.57E-03
any	EAS	5,225	0.099	0.063-0.147	1.47E-69	3.68E-69	59.052	65.071	3.98E-03	9.96E-03
BCC	EAS	5,225	0.067	0.031-0.128	1.28E-43	3.01E-43	58.521	65.693	5.57E-02	1.11E-01
melanoma	EAS	5,225	0.085	0.023-0.218	3.69E-15	6.72E-15	53.937	62.540	7.54E-02	1.38E-01
other	EAS	5,225	0.091	0.037-0.188	8.90E-24	1.87E-23	56.381	61.690	4.43E-01	6.56E-01
SCC	EAS	5,225	0.116	0.05-0.229	8.86E-20	1.77E-19	59.512	67.462	1.48E-02	3.48E-02
melanoma	EUR	116,847	6.698	5.881-7.653	7.09E-271	3.15E-270	62.351	63.649	9.15E-02	1.46E-01
SCC	EUR	116,847	6.502	5.846-7.245	0.00E+00	0.00E+00	67.479	67.203	6.80E-01	9.38E-01
other	EUR	116,847	6.009	5.449-6.638	0.00E+00	0.00E+00	61.619	62.422	3.27E-01	5.03E-01
any	EUR	116,847	6.812	6.43-7.222	0.00E+00	0.00E+00	65.047	65.101	9.09E-02	1.46E-01
BCC	EUR	116,847	8.489	7.801-9.251	0.00E+00	0.00E+00	65.477	67.626	2.24E-05	1.05E-04
melanoma	MID	505	0.450	0.054-1.637	3.39E-01	3.87E-01	65.208	62.526	9.15E-01	1.00E+00
SCC	MID	505	0.153	0.004-0.855	2.59E-02	3.24E-02	48.915	67.441	3.48E-05	1.39E-04
other	MID	505	0.275	0.033-0.999	5.60E-02	6.79E-02	43.799	61.696	2.36E-05	1.05E-04
BCC	MID	505	0.159	0.019-0.576	7.23E-04	1.03E-03	53.963	65.686	8.56E-02	1.46E-01
any	MID	505	0.307	0.123-0.638	2.81E-04	4.16E-04	48.915	65.052	3.26E-03	8.69E-03
other	OTH	17,765	1.222	1.081-1.377	1.23E-03	1.70E-03	64.860	61.367	2.28E-05	1.05E-04
melanoma	OTH	17,765	1.198	1.023-1.396	2.34E-02	3.02E-02	65.690	62.152	8.68E-04	2.48E-03
any	OTH	17,765	1.176	1.095-1.263	9.32E-06	1.49E-05	67.690	64.782	2.64E-12	1.06E-10
BCC	OTH	17,765	1.094	0.992-1.204	6.85E-02	8.06E-02	68.474	65.438	2.31E-08	1.54E-07
SCC	OTH	17,765	1.183	1.038-1.344	1.12E-02	1.50E-02	69.926	67.151	8.58E-05	3.12E-04
SCC	SAS	2,128	0.108	0.022-0.316	9.26E-09	1.54E-08	66.556	67.441	8.32E-01	1.00E+00
other	SAS	2,128	0.097	0.02-0.284	5.35E-10	9.30E-10	46.425	61.690	7.61E-02	1.38E-01
BCC	SAS	2,128	0.056	0.012-0.164	7.62E-19	1.45E-18	72.058	65.682	9.28E-01	1.00E+00
any	SAS	2,128	0.113	0.056-0.202	1.80E-27	4.00E-27	64.241	65.047	7.23E-01	9.65E-01
melanoma	SAS	2,128	0.159	0.033-0.465	1.83E-05	2.82E-05	46.425	62.536	6.28E-01	8.97E-01

Table S3: Comparing genotype PC values between skin cancer patients and healthy individuals

For each genetic ancestry, shown are the comparison between the distributions of each genotype PC level between skin cancer patients and healthy individuals. Shown are: the mean PC value for healthy individuals and skin cancer patients, the two-sided p-value (t test) and adjusted p-value (Benjamini-Hochberg).

Ancestry	PC	Mean (healthy)	Mean (cancer)	p-value (t test)	Adjusted p-value (Benjamini-Hochberg)
AFR	pc1	-0.24827931	-0.24276555	0.11291886	0.36134035
EAS	pc1	0.09844291	0.09884036	0.30466059	0.64994259
AMR	pc1	0.06448239	0.06120198	0.14395834	0.41878788
MID	pc1	0.05480666	0.07145187	0.10569485	0.3599207
SAS	pc1	0.08542451	0.08623759	0.29984933	0.64994259
OTH	pc1	0.03312291	0.07965884	3.952E-221	1.897E-219
AFR	pc2	0.01049325	0.01243827	0.10049521	0.35731632
EAS	pc2	-0.16511779	-0.16659464	0.24447937	0.62075592
AMR	pc2	0.02606956	0.05116428	7.174E-20	7.6523E-19
MID	pc2	0.10229471	0.10968445	0.01981856	0.09059913
SAS	pc2	0.02443447	0.03005856	0.3503094	0.68632046
OTH	pc2	0.08169282	0.10998007	1.484E-250	1.425E-248
AFR	pc3	0.00119862	0.00090553	0.31415817	0.65563444
EAS	pc3	-0.04101257	-0.04084692	0.90407639	0.97518352
AMR	pc3	0.10404265	0.07340969	2.704E-17	2.5959E-16
MID	pc3	-0.02341887	-0.02415284	0.76776677	0.9362324
SAS	pc3	-0.04134547	-0.04061936	0.69596118	0.9362324
OTH	pc3	-0.00551113	-0.01667714	1.887E-148	4.53E-147
AFR	pc4	0.00731423	0.00801619	0.10872604	0.3599207
EAS	pc4	0.03699554	0.03724127	0.79628628	0.9362324
AMR	pc4	0.00692544	0.0162517	3.136E-17	2.7369E-16
MID	pc4	0.00148562	-0.00020544	0.79860018	0.9362324
SAS	pc4	-0.09199914	-0.09265398	0.9020911	0.97518352
OTH	pc4	0.02164089	0.0199309	1.2115E-07	7.7538E-07
AFR	pc5	0.01639693	0.01644757	0.94786308	0.97843931
EAS	pc5	0.00010824	-3.9597E-05	0.34563093	0.68632046
AMR	pc5	-0.0012486	-0.00120056	0.79969851	0.9362324

MID	pc5	-0.00535089	-0.00494186	0.86076846	0.97216203
SAS	pc5	-0.00047266	-0.00029445	0.55365313	0.90582142
OTH	pc5	0.00047633	-0.00141093	3.7465E-56	5.1381E-55
AFR	pc6	-0.00278771	-0.00258889	0.82573308	0.95506477
EAS	pc6	-0.00212861	-0.00213904	0.96945547	0.98036616
AMR	pc6	-0.00508371	-0.0063913	0.00014435	0.00072936
MID	pc6	-0.05519538	-0.04749336	0.0302221	0.1261444
SAS	pc6	0.00743823	0.00711789	0.59482027	0.92101204
OTH	pc6	-0.01124817	-0.0217722	2.904E-121	5.576E-120
AFR	pc7	0.00948295	0.01002516	0.36068773	0.69252045
EAS	pc7	-0.00308234	-0.00333781	0.47153741	0.8705306
AMR	pc7	-0.00541805	-0.0075767	4.4507E-07	2.6704E-06
MID	pc7	-0.06363909	-0.06123165	0.1360499	0.41878788
SAS	pc7	0.00658718	0.00574161	0.27532075	0.62930456
OTH	pc7	-0.01214269	-0.02897404	7.965E-161	2.549E-159
AFR	pc8	0.00566066	0.00548964	0.70333922	0.9362324
EAS	pc8	0.00063848	0.00088613	0.16956043	0.46508003
AMR	pc8	0.00099228	0.0012578	0.05244573	0.20978293
MID	pc8	0.01110601	0.01152496	0.68374002	0.9362324
SAS	pc8	-0.00113843	-0.00049265	0.0653672	0.25101004
OTH	pc8	0.00308186	0.00544012	4.8072E-95	7.6915E-94
AFR	pc9	0.0009559	0.00068736	0.5406736	0.90582142
EAS	pc9	0.00037412	0.00014865	0.1404609	0.41878788
AMR	pc9	0.00012012	0.00015914	0.72006616	0.9362324
MID	pc9	0.00358997	0.0022746	0.32280366	0.65934365
SAS	pc9	-0.00039962	-0.00015163	0.25950027	0.62075592
OTH	pc9	0.00083976	0.00162788	3.1663E-47	3.7996E-46
AFR	pc10	0.00169751	0.00156403	0.74384326	0.9362324
EAS	pc10	8.0883E-05	2.2551E-05	0.70106554	0.9362324
AMR	pc10	0.00014767	0.00013358	0.90060312	0.97518352
MID	pc10	-0.00047239	0.00033784	0.01482556	0.07116271
SAS	pc10	0.00027419	0.00013123	0.52631095	0.90224734
OTH	pc10	4.2881E-05	-0.0003456	6.0418E-16	4.4616E-15

AFR	pc11	8.2356E-05	0.00033004	0.56953859	0.90582142
EAS	pc11	-8.918E-05	-5.2421E-05	0.78933324	0.9362324
AMR	pc11	0.00012187	8.8751E-05	0.78256774	0.9362324
MID	pc11	0.00042255	0.00082825	0.51125164	0.90224734
SAS	pc11	-0.00016055	-0.00028773	0.65925314	0.9362324
OTH	pc11	7.0867E-05	0.00035872	1.8798E-09	1.289E-08
AFR	pc12	0.00067113	0.00090967	0.57557403	0.90582142
EAS	pc12	7.2541E-05	0.00023652	0.26511451	0.62075592
AMR	pc12	-9.8935E-08	6.4054E-05	0.56756496	0.90582142
MID	pc12	0.00113725	0.00090274	0.50926107	0.90224734
SAS	pc12	-0.0001318	0.00020296	0.14850621	0.41931165
OTH	pc12	0.00023682	0.00021621	0.64453037	0.9362324
AFR	pc13	-0.00104643	-0.00056685	0.24759241	0.62075592
EAS	pc13	-6.0216E-05	-5.818E-05	0.99054214	0.99054214
AMR	pc13	-9.1196E-05	-8.2255E-05	0.94246277	0.97843931
MID	pc13	-6.6009E-05	7.8939E-05	0.75164941	0.9362324
SAS	pc13	-2.0655E-05	-0.00030001	0.08776705	0.32406296
OTH	pc13	-0.00014796	8.8192E-05	2.9073E-06	1.6417E-05
AFR	pc14	0.00104605	0.00062965	0.28853612	0.64417367
EAS	pc14	5.9429E-05	0.00011257	0.67238463	0.9362324
AMR	pc14	0.00017526	0.00031476	0.25594993	0.62075592
MID	pc14	-0.00086599	-0.00108597	0.74233155	0.9362324
SAS	pc14	0.00025777	0.00027923	0.91725874	0.97840932
OTH	pc14	-0.00014087	-0.00051098	1.9374E-16	1.5499E-15
AFR	pc15	-0.00087385	-0.00085919	0.97015401	0.98036616
EAS	pc15	-0.00014214	-0.00018643	0.77367065	0.9362324
AMR	pc15	-0.00016299	-0.0001423	0.85751677	0.97216203
MID	pc15	0.00010657	-2.0188E-05	0.89215964	0.97518352
SAS	pc15	1.7589E-05	0.00014333	0.6320609	0.9362324
OTH	pc15	-0.00010059	0.00010291	7.2353E-05	0.00038588
AFR	pc16	3.7803E-05	-0.00012456	0.70383895	0.9362324

EAS	pc16	8.2101E-06	0.00010965	0.52031984	0.90224734
AMR	pc16	-5.3475E-05	0.00010654	0.196302	0.52347199
MID	pc16	-0.00049128	-0.00044275	0.9393005	0.97843931
SAS	pc16	9.7443E-06	0.00024422	0.43187636	0.81294373
OTH	pc16	-5.0831E-05	-0.00015274	0.02260796	0.09865294

Table S4: Factors that influence skin cancer risk

For each covariate in Figure 4 shown are the effect sizes, their standard errors and two-sided p-values for each logistic regression model (*glm* function in R). Which populations (All participants, EUR, and non-EUR) and sex (all (both sexes), males, and females) the model included are shown. The last column describes whether the logistic regression model was performed individually for each covariate ("single") or a combined logistic regression using all the covariates ("combined").

Covariate	Sex	Population	Beta	SE	p-value	Model
cancer history	all	ALL	1.834	0.022	0.00E+00	single
age at diagnosis	all	ALL	0.173	0.004	0.00E+00	single
sex	all	ALL	0.474	0.020	2.95E-120	single
annual income	all	ALL	0.216	0.004	0.00E+00	single
insurance	all	ALL	2.381	0.114	4.55E-97	single
education	all	ALL	0.451	0.009	0.00E+00	single
living	all	ALL	-1.402	0.047	4.10E-193	single
alcohol daily	all	ALL	-0.063	0.012	1.07E-07	single
alcohol yearly	all	ALL	0.259	0.007	0.00E+00	single
smoking daily	all	ALL	0.064	0.004	2.49E-71	single
smoking yearly	all	ALL	0.057	0.004	4.82E-57	single
latitude	all	ALL	0.130	0.004	1.40E-271	single
longitude	all	ALL	0.092	0.004	8.91E-143	single
hispanic	all	ALL	-1.870	0.052	1.90E-287	single
african	all	ALL	-2.979	0.084	3.43E-274	single
european	all	ALL	2.656	0.041	0.00E+00	single
admixed	all	ALL	-1.337	0.152	1.28E-18	single
asian	all	ALL	-2.342	0.175	5.13E-41	single
middle eastern	all	ALL	-1.673	0.303	3.38E-08	single
pacific	all	ALL	-2.295	1.001	2.19E-02	single
pc1	all	ALL	0.267	0.004	0.00E+00	single
pc2	all	ALL	0.309	0.004	0.00E+00	single
pc3	all	ALL	-0.224	0.004	0.00E+00	single
pc4	all	ALL	0.280	0.004	0.00E+00	single
pc5	all	ALL	-0.029	0.004	1.66E-16	single
pc6	all	ALL	0.202	0.004	0.00E+00	single
pc7	all	ALL	0.176	0.004	0.00E+00	single
pc8	all	ALL	-0.197	0.004	0.00E+00	single
pc9	all	ALL	-0.070	0.004	7.79E-86	single
pc10	all	ALL	-0.065	0.004	6.79E-75	single
pc11	all	ALL	0.009	0.004	1.49E-02	single

pc12	all	ALL	-0.044	0.004	1.07E-34	single
pc13	all	ALL	0.025	0.004	1.30E-12	single
pc14	all	ALL	-0.050	0.004	1.94E-44	single
pc15	all	ALL	0.044	0.004	6.45E-36	single
pc16	all	ALL	0.018	0.004	2.45E-07	single
avanafil	all	ALL	1.038	0.531	5.07E-02	single
sildenafil	all	ALL	1.244	0.062	8.77E-90	single
tadalafil	all	ALL	1.274	0.075	5.25E-64	single
cancer history	all	EUR	1.535	0.024	0.00E+00	single
age at diagnosis	all	EUR	0.075	0.004	1.29E-82	single
sex	all	EUR	0.471	0.022	2.19E-99	single
annual income	all	EUR	0.104	0.005	3.89E-91	single
insurance	all	EUR	1.751	0.137	3.86E-37	single
education	all	EUR	0.219	0.010	6.30E-97	single
living	all	EUR	-1.147	0.054	3.71E-100	single
alcohol daily	all	EUR	-0.152	0.015	5.73E-24	single
alcohol yearly	all	EUR	0.125	0.008	1.23E-60	single
smoking daily	all	EUR	0.026	0.004	2.03E-11	single
smoking yearly	all	EUR	0.025	0.004	2.27E-10	single
latitude	all	EUR	0.077	0.004	1.63E-86	single
longitude	all	EUR	0.082	0.004	5.04E-97	single
hispanic	all	EUR	-0.516	0.151	6.56E-04	single
african	all	EUR	-10.057	63.681	8.75E-01	single
european	all	EUR	0.672	0.122	4.04E-08	single
admixed	all	EUR	-0.798	0.212	1.73E-04	single
asian	all	EUR	-9.057	69.637	8.97E-01	single
middle eastern	all	EUR	-11.057	70.286	8.75E-01	single
pacific	all	EUR	0.111	1.045	9.15E-01	single
pc1	all	EUR	0.052	0.004	7.10E-41	single
pc2	all	EUR	0.042	0.004	7.22E-27	single
pc3	all	EUR	0.022	0.004	1.41E-08	single
pc4	all	EUR	0.033	0.004	1.89E-17	single
pc5	all	EUR	0.018	0.004	2.76E-06	single
pc6	all	EUR	0.031	0.004	1.60E-15	single
pc7	all	EUR	0.024	0.004	5.37E-10	single
pc8	all	EUR	-0.016	0.004	2.14E-05	single
pc9	all	EUR	-0.007	0.004	7.52E-02	single
pc10	all	EUR	0.006	0.004	9.89E-02	single
pc11	all	EUR	-0.010	0.004	9.18E-03	single
pc12	all	EUR	-0.007	0.004	6.12E-02	single

pc13	all	EUR	-0.010	0.004	8.20E-03	single
pc14	all	EUR	0.004	0.004	2.90E-01	single
pc15	all	EUR	-0.003	0.004	4.00E-01	single
pc16	all	EUR	0.004	0.004	2.56E-01	single
avanafil	all	EUR	0.775	0.626	2.16E-01	single
sildenafil	all	EUR	1.046	0.069	1.01E-51	single
tadalafil	all	EUR	1.095	0.084	1.21E-38	single
cancer history	male	EUR	1.593	0.034	0.00E+00	single
age at diagnosis	male	EUR	0.061	0.006	1.60E-28	single
annual income	male	EUR	0.102	0.007	7.35E-47	single
insurance	male	EUR	1.564	0.152	6.46E-25	single
education	male	EUR	0.262	0.015	2.35E-71	single
living	male	EUR	-1.306	0.076	6.42E-66	single
alcohol daily	male	EUR	-0.193	0.019	1.83E-24	single
alcohol yearly	male	EUR	0.087	0.010	2.61E-17	single
smoking daily	male	EUR	0.024	0.006	8.96E-06	single
smoking yearly	male	EUR	0.014	0.006	1.16E-02	single
latitude	male	EUR	0.080	0.006	1.22E-46	single
longitude	male	EUR	0.073	0.006	1.63E-39	single
hispanic	male	EUR	-0.339	0.200	9.02E-02	single
african	male	EUR	-10.318	93.735	9.12E-01	single
european	male	EUR	0.487	0.162	2.65E-03	single
admixed	male	EUR	-0.610	0.286	3.27E-02	single
asian	male	EUR	-9.318	88.084	9.16E-01	single
middle eastern	male	EUR	-10.319	67.698	8.79E-01	single
pacific	male	EUR	0.862	1.118	4.41E-01	single
pc1	male	EUR	0.057	0.006	6.17E-25	single
pc2	male	EUR	0.047	0.006	3.05E-17	single
pc3	male	EUR	0.022	0.006	5.86E-05	single
pc4	male	EUR	0.032	0.006	4.68E-09	single
pc5	male	EUR	0.024	0.006	1.41E-05	single
pc6	male	EUR	0.036	0.006	6.94E-11	single
pc7	male	EUR	0.030	0.006	7.19E-08	single
pc8	male	EUR	-0.021	0.006	9.72E-05	single
pc9	male	EUR	-0.010	0.006	6.02E-02	single
pc10	male	EUR	0.007	0.006	2.11E-01	single
pc11	male	EUR	-0.019	0.006	6.41E-04	single
pc12	male	EUR	-0.012	0.006	2.36E-02	single
pc13	male	EUR	-0.016	0.006	4.73E-03	single
pc14	male	EUR	0.007	0.006	2.15E-01	single

pc15	male	EUR	-0.003	0.006	6.26E-01	single
pc16	male	EUR	0.012	0.006	2.42E-02	single
avanafil	male	EUR	0.514	0.626	4.12E-01	single
sildenafil	male	EUR	0.808	0.070	1.13E-30	single
tadalafil	male	EUR	0.856	0.085	1.22E-23	single
cancer history	female	EUR	1.461	0.034	0.00E+00	single
age at diagnosis	female	EUR	0.071	0.005	8.52E-38	single
annual income	female	EUR	0.098	0.007	5.28E-40	single
insurance	female	EUR	2.580	0.334	1.20E-14	single
education	female	EUR	0.174	0.015	1.94E-31	single
living	female	EUR	-1.036	0.077	1.07E-41	single
alcohol daily	female	EUR	-0.139	0.024	6.70E-09	single
alcohol yearly	female	EUR	0.141	0.011	1.80E-36	single
smoking daily	female	EUR	0.016	0.005	3.83E-03	single
smoking yearly	female	EUR	0.024	0.005	1.24E-05	single
latitude	female	EUR	0.079	0.006	8.40E-47	single
longitude	female	EUR	0.098	0.006	4.14E-69	single
hispanic	female	EUR	-0.715	0.233	2.20E-03	single
african	female	EUR	-9.848	86.784	9.10E-01	single
european	female	EUR	0.875	0.189	3.63E-06	single
admixed	female	EUR	-0.974	0.321	2.38E-03	single
asian	female	EUR	-7.847	68.974	9.09E-01	single
middle eastern	female	EUR	-10.848	90.480	9.05E-01	single
pacific	female	EUR	-8.847	74.444	9.05E-01	single
pc1	female	EUR	0.046	0.005	5.63E-17	single
pc2	female	EUR	0.035	0.005	1.08E-10	single
pc3	female	EUR	0.021	0.005	8.87E-05	single
pc4	female	EUR	0.033	0.005	1.12E-09	single
pc5	female	EUR	0.013	0.005	1.42E-02	single
pc6	female	EUR	0.026	0.005	2.08E-06	single
pc7	female	EUR	0.018	0.005	7.07E-04	single
pc8	female	EUR	-0.012	0.005	2.90E-02	single
pc9	female	EUR	-0.005	0.005	3.92E-01	single
pc10	female	EUR	0.005	0.005	3.48E-01	single
pc11	female	EUR	-0.001	0.005	8.73E-01	single
pc12	female	EUR	-0.003	0.005	5.27E-01	single
pc13	female	EUR	-0.004	0.005	4.52E-01	single
pc14	female	EUR	0.001	0.005	8.63E-01	single
pc15	female	EUR	-0.003	0.005	5.41E-01	single
pc16	female	EUR	-0.004	0.005	5.13E-01	single

cancer history	all	not EUR	2.482	0.055	0.00E+00	single
age at diagnosis	all	not EUR	0.261	0.011	5.89E-127	single
sex	all	not EUR	0.497	0.055	7.62E-20	single
annual income	all	not EUR	0.330	0.012	2.04E-171	single
insurance	all	not EUR	2.015	0.206	1.41E-22	single
education	all	not EUR	0.582	0.026	1.32E-114	single
living	all	not EUR	-1.138	0.101	2.14E-29	single
alcohol daily	all	not EUR	-0.072	0.030	1.59E-02	single
alcohol yearly	all	not EUR	0.233	0.018	1.11E-36	single
smoking daily	all	not EUR	0.070	0.010	2.59E-13	single
smoking yearly	all	not EUR	0.064	0.010	2.80E-11	single
latitude	all	not EUR	0.126	0.010	7.29E-37	single
longitude	all	not EUR	0.153	0.010	6.64E-51	single
hispanic	all	not EUR	-0.646	0.062	3.51E-25	single
african	all	not EUR	-1.850	0.088	2.81E-97	single
european	all	not EUR	2.940	0.056	0.00E+00	single
admixed	all	not EUR	-0.544	0.221	1.37E-02	single
asian	all	not EUR	-1.010	0.177	1.10E-08	single
middle eastern	all	not EUR	-0.282	0.304	3.54E-01	single
pacific	all	not EUR	-11.140	101.313	9.12E-01	single
pc1	all	not EUR	0.256	0.011	1.08E-127	single
pc2	all	not EUR	0.525	0.023	2.68E-116	single
pc3	all	not EUR	-0.268	0.012	1.56E-118	single
pc4	all	not EUR	0.245	0.011	8.45E-115	single
pc5	all	not EUR	-0.274	0.011	1.06E-134	single
pc6	all	not EUR	-0.376	0.015	1.64E-129	single
pc7	all	not EUR	-0.444	0.018	2.87E-130	single
pc8	all	not EUR	0.134	0.010	5.80E-42	single
pc9	all	not EUR	0.114	0.010	7.32E-32	single
pc10	all	not EUR	-0.095	0.010	8.17E-23	single
pc11	all	not EUR	0.031	0.010	9.22E-04	single
pc12	all	not EUR	0.004	0.009	7.10E-01	single
pc13	all	not EUR	0.049	0.010	2.69E-07	single
pc14	all	not EUR	-0.091	0.010	2.71E-21	single
pc15	all	not EUR	0.045	0.010	2.17E-06	single
pc16	all	not EUR	-0.013	0.009	1.75E-01	single
avanafil	all	not EUR	1.789	1.035	8.40E-02	single
sildenafil	all	not EUR	1.556	0.154	5.28E-24	single
tadalafil	all	not EUR	1.537	0.190	5.17E-16	single

cancer history	male	not EUR	2.624	0.078	5.46E-244	single
age at diagnosis	male	not EUR	0.288	0.016	1.07E-75	single
annual income	male	not EUR	0.370	0.016	1.16E-120	single
insurance	male	not EUR	2.092	0.261	1.25E-15	single
education	male	not EUR	0.737	0.038	2.73E-82	single
living	male	not EUR	-1.502	0.143	1.18E-25	single
alcohol daily	male	not EUR	-0.137	0.038	3.59E-04	single
alcohol yearly	male	not EUR	0.194	0.025	5.69E-15	single
smoking daily	male	not EUR	0.056	0.013	3.03E-05	single
smoking yearly	male	not EUR	0.025	0.013	5.97E-02	single
latitude	male	not EUR	0.120	0.014	6.32E-18	single
longitude	male	not EUR	0.151	0.014	6.54E-26	single
hispanic	male	not EUR	-0.577	0.092	3.67E-10	single
african	male	not EUR	-2.332	0.139	2.36E-63	single
european	male	not EUR	3.075	0.082	2.15E-304	single
admixed	male	not EUR	-0.823	0.381	3.08E-02	single
asian	male	not EUR	-1.174	0.262	7.23E-06	single
middle eastern	male	not EUR	-0.440	0.412	2.86E-01	single
pacific	male	not EUR	-11.415	148.385	9.39E-01	single
pc1	male	not EUR	0.322	0.016	5.39E-93	single
pc2	male	not EUR	0.662	0.049	5.30E-41	single
pc3	male	not EUR	-0.287	0.017	1.10E-65	single
pc4	male	not EUR	0.293	0.016	2.37E-76	single
pc5	male	not EUR	-0.342	0.017	1.01E-92	single
pc6	male	not EUR	-0.431	0.026	5.87E-62	single
pc7	male	not EUR	-0.537	0.035	4.15E-54	single
pc8	male	not EUR	0.130	0.014	3.36E-21	single
pc9	male	not EUR	0.123	0.014	1.79E-19	single
pc10	male	not EUR	-0.122	0.014	4.36E-19	single
pc11	male	not EUR	0.029	0.013	2.75E-02	single
pc12	male	not EUR	-0.002	0.013	8.90E-01	single
pc13	male	not EUR	0.062	0.013	3.47E-06	single
pc14	male	not EUR	-0.112	0.014	1.54E-16	single
pc15	male	not EUR	0.053	0.013	7.88E-05	single
pc16	male	not EUR	-0.010	0.013	4.53E-01	single
avanafil	male	not EUR	1.516	1.036	1.43E-01	single
sildenafil	male	not EUR	1.312	0.157	5.57E-17	single
tadalafil	male	not EUR	1.286	0.192	1.95E-11	single
cancer history	female	not EUR	2.364	0.079	9.46E-196	single

age at diagnosis	female	not EUR	0.222	0.015	4.64E-50	single
annual income	female	not EUR	0.269	0.018	2.97E-53	single
insurance	female	not EUR	2.074	0.336	6.72E-10	single
education	female	not EUR	0.431	0.033	5.07E-38	single
living	female	not EUR	-0.857	0.142	1.77E-09	single
alcohol daily	female	not EUR	-0.058	0.047	2.20E-01	single
alcohol yearly	female	not EUR	0.231	0.028	1.17E-16	single
smoking daily	female	not EUR	0.072	0.014	1.54E-07	single
smoking yearly	female	not EUR	0.081	0.014	4.98E-09	single
latitude	female	not EUR	0.132	0.014	2.40E-20	single
longitude	female	not EUR	0.155	0.015	1.56E-26	single
hispanic	female	not EUR	-0.618	0.085	4.17E-13	single
african	female	not EUR	-1.482	0.115	8.16E-38	single
european	female	not EUR	2.763	0.079	1.82E-265	single
admixed	female	not EUR	-0.316	0.271	2.44E-01	single
asian	female	not EUR	-0.869	0.239	2.84E-04	single
middle eastern	female	not EUR	-0.184	0.451	6.84E-01	single
pacific	female	not EUR	-10.917	138.660	9.37E-01	single
pc1	female	not EUR	0.204	0.015	4.06E-44	single
pc2	female	not EUR	0.418	0.024	1.97E-67	single
pc3	female	not EUR	-0.240	0.016	3.27E-51	single
pc4	female	not EUR	0.204	0.015	3.38E-43	single
pc5	female	not EUR	-0.224	0.015	1.05E-49	single
pc6	female	not EUR	-0.324	0.019	1.28E-63	single
pc7	female	not EUR	-0.368	0.021	3.66E-67	single
pc8	female	not EUR	0.129	0.014	3.28E-20	single
pc9	female	not EUR	0.096	0.014	3.34E-12	single
pc10	female	not EUR	-0.067	0.014	9.37E-07	single
pc11	female	not EUR	0.032	0.014	1.68E-02	single
pc12	female	not EUR	0.009	0.014	5.10E-01	single
pc13	female	not EUR	0.034	0.014	1.24E-02	single
pc14	female	not EUR	-0.071	0.014	2.47E-07	single
pc15	female	not EUR	0.039	0.014	3.98E-03	single
pc16	female	not EUR	-0.017	0.014	2.16E-01	single
cancer history	all	ALL	1.439	0.025	0.00E+00	combined
age at diagnosis	all	ALL	-0.002	0.001	4.91E-02	combined
sex	all	ALL	0.440	0.023	2.03E-79	combined
annual income	all	ALL	0.029	0.006	1.38E-06	combined
insurance	all	ALL	0.867	0.122	1.23E-12	combined

education	all	ALL	0.117	0.013	1.12E-20	combined
living	all	ALL	-0.635	0.053	5.94E-33	combined
alcohol daily	all	ALL	-0.369	0.022	2.10E-62	combined
alcohol yearly	all	ALL	0.155	0.010	4.15E-54	combined
smoking daily	all	ALL	0.010	0.001	1.94E-14	combined
smoking yearly	all	ALL	0.005	0.001	1.28E-05	combined
latitude	all	ALL	0.010	0.003	3.53E-04	combined
longitude	all	ALL	0.010	0.001	3.98E-30	combined
hispanic	all	ALL	0.157	0.988	8.74E-01	combined
african	all	ALL	0.305	1.018	7.64E-01	combined
european	all	ALL	0.778	0.983	4.28E-01	combined
admixed	all	ALL	-0.168	0.989	8.65E-01	combined
asian	all	ALL	-0.411	1.001	6.81E-01	combined
middle eastern	all	ALL	-1.180	1.056	2.64E-01	combined
pc1	all	ALL	4.320	0.968	8.14E-06	combined
pc2	all	ALL	6.017	1.270	2.15E-06	combined
pc3	all	ALL	-3.888	1.537	1.14E-02	combined
pc4	all	ALL	5.679	1.845	2.09E-03	combined
pc5	all	ALL	-1.851	6.788	7.85E-01	combined
pc6	all	ALL	13.006	6.539	4.67E-02	combined
pc7	all	ALL	-6.251	5.182	2.28E-01	combined
pc8	all	ALL	26.400	9.461	5.27E-03	combined
pc9	all	ALL	-4.013	10.446	7.01E-01	combined
pc10	all	ALL	8.486	10.494	4.19E-01	combined
pc11	all	ALL	-7.544	10.330	4.65E-01	combined
pc12	all	ALL	-4.171	10.482	6.91E-01	combined
pc13	all	ALL	-8.744	10.414	4.01E-01	combined
pc14	all	ALL	-5.218	10.215	6.09E-01	combined
pc15	all	ALL	1.018	10.302	9.21E-01	combined
pc16	all	ALL	12.073	10.330	2.43E-01	combined
avanafil	all	ALL	-0.740	0.735	3.14E-01	combined
sildenafil	all	ALL	0.391	0.074	1.09E-07	combined
tadalafil	all	ALL	0.335	0.089	1.53E-04	combined
cancer history	all	EUR	1.394	0.027	0.00E+00	combined
age at diagnosis	all	EUR	-0.002	0.001	1.42E-02	combined
sex	all	EUR	0.446	0.025	2.14E-68	combined
annual income	all	EUR	0.024	0.007	2.31E-04	combined
insurance	all	EUR	0.962	0.145	3.31E-11	combined
education	all	EUR	0.116	0.014	3.34E-17	combined
living	all	EUR	-0.715	0.061	7.79E-32	combined

alcohol daily	all	EUR	-0.388	0.024	1.86E-57	combined
alcohol yearly	all	EUR	0.167	0.011	1.70E-53	combined
smoking daily	all	EUR	0.009	0.001	3.03E-11	combined
smoking yearly	all	EUR	0.005	0.001	2.75E-05	combined
latitude	all	EUR	0.009	0.003	3.23E-03	combined
longitude	all	EUR	0.010	0.001	8.58E-27	combined
hispanic	all	EUR	-1.154	1.074	2.83E-01	combined
african	all	EUR	-11.754	142.630	9.34E-01	combined
european	all	EUR	-0.882	1.058	4.05E-01	combined
admixed	all	EUR	-1.380	1.083	2.02E-01	combined
asian	all	EUR	-11.169	190.219	9.53E-01	combined
middle eastern	all	EUR	-11.144	80.310	8.90E-01	combined
pc1	all	EUR	19.059	6.008	1.51E-03	combined
pc2	all	EUR	18.464	4.159	9.03E-06	combined
pc3	all	EUR	9.537	5.862	1.04E-01	combined
pc4	all	EUR	0.943	3.728	8.00E-01	combined
pc5	all	EUR	-14.480	14.188	3.07E-01	combined
pc6	all	EUR	32.308	10.678	2.48E-03	combined
pc7	all	EUR	-15.749	8.045	5.03E-02	combined
pc8	all	EUR	34.090	14.296	1.71E-02	combined
pc9	all	EUR	9.635	15.127	5.24E-01	combined
pc10	all	EUR	16.208	15.190	2.86E-01	combined
pc11	all	EUR	-19.974	14.923	1.81E-01	combined
pc12	all	EUR	-8.937	15.049	5.53E-01	combined
pc13	all	EUR	-31.225	14.785	3.47E-02	combined
pc14	all	EUR	4.190	14.432	7.72E-01	combined
pc15	all	EUR	-10.503	14.652	4.73E-01	combined
pc16	all	EUR	12.987	14.595	3.74E-01	combined
avanafil	all	EUR	-0.626	0.766	4.14E-01	combined
sildenafil	all	EUR	0.373	0.081	4.53E-06	combined
tadalafil	all	EUR	0.357	0.098	2.66E-04	combined
cancer history	male	EUR	1.467	0.039	0.00E+00	combined
age at diagnosis	male	EUR	-0.005	0.001	1.37E-04	combined
annual income	male	EUR	0.008	0.009	3.95E-01	combined
insurance	male	EUR	0.656	0.162	4.85E-05	combined
education	male	EUR	0.162	0.020	3.40E-16	combined
living	male	EUR	-0.782	0.087	2.14E-19	combined
alcohol daily	male	EUR	-0.351	0.031	1.05E-29	combined
alcohol yearly	male	EUR	0.141	0.015	4.61E-21	combined

smoking daily	male	EUR	0.010	0.002	9.69E-10	combined
smoking yearly	male	EUR	0.003	0.002	3.39E-02	combined
latitude	male	EUR	0.010	0.004	2.61E-02	combined
longitude	male	EUR	0.007	0.001	1.96E-08	combined
hispanic	male	EUR	-1.571	1.178	1.82E-01	combined
african	male	EUR	-12.766	184.447	9.45E-01	combined
european	male	EUR	-1.541	1.154	1.82E-01	combined
admixed	male	EUR	-1.695	1.190	1.54E-01	combined
asian	male	EUR	-11.877	254.051	9.63E-01	combined
middle eastern	male	EUR	-12.223	136.382	9.29E-01	combined
pc1	male	EUR	22.998	8.581	7.36E-03	combined
pc2	male	EUR	21.896	5.868	1.91E-04	combined
pc3	male	EUR	6.040	8.318	4.68E-01	combined
pc4	male	EUR	-0.997	5.274	8.50E-01	combined
pc5	male	EUR	-20.902	20.079	2.98E-01	combined
pc6	male	EUR	39.423	15.235	9.67E-03	combined
pc7	male	EUR	-23.047	11.458	4.43E-02	combined
pc8	male	EUR	25.232	20.363	2.15E-01	combined
pc9	male	EUR	0.640	21.424	9.76E-01	combined
pc10	male	EUR	10.749	21.522	6.17E-01	combined
pc11	male	EUR	-53.368	21.147	1.16E-02	combined
pc12	male	EUR	-26.159	21.390	2.21E-01	combined
pc13	male	EUR	-60.061	21.018	4.27E-03	combined
pc14	male	EUR	21.451	20.435	2.94E-01	combined
pc15	male	EUR	-13.823	20.762	5.06E-01	combined
pc16	male	EUR	28.979	20.650	1.61E-01	combined
avanafil	male	EUR	-0.618	0.767	4.20E-01	combined
sildenafil	male	EUR	0.400	0.082	1.13E-06	combined
tadalafil	male	EUR	0.395	0.099	6.30E-05	combined
cancer history	female	EUR	1.321	0.039	1.18E-249	combined
age at diagnosis	female	EUR	0.000	0.001	9.22E-01	combined
annual income	female	EUR	0.040	0.009	1.41E-05	combined
insurance	female	EUR	1.825	0.352	2.24E-07	combined
education	female	EUR	0.074	0.019	1.22E-04	combined
living	female	EUR	-0.653	0.085	2.19E-14	combined
alcohol daily	female	EUR	-0.440	0.039	2.30E-29	combined
alcohol yearly	female	EUR	0.197	0.016	9.18E-36	combined
smoking daily	female	EUR	0.007	0.002	1.07E-03	combined
smoking yearly	female	EUR	0.007	0.002	1.08E-04	combined

latitude	female	EUR	0.009	0.005	5.77E-02	combined
longitude	female	EUR	0.013	0.001	1.22E-21	combined
hispanic	female	EUR	9.407	209.194	9.64E-01	combined
african	female	EUR	-0.215	307.334	9.99E-01	combined
european	female	EUR	9.972	209.194	9.62E-01	combined
admixed	female	EUR	9.058	209.195	9.65E-01	combined
asian	female	EUR	-0.230	354.824	9.99E-01	combined
middle eastern	female	EUR	0.025	232.318	1.00E+00	combined
pc1	female	EUR	16.214	8.432	5.45E-02	combined
pc2	female	EUR	14.955	5.908	1.14E-02	combined
pc3	female	EUR	12.959	8.280	1.18E-01	combined
pc4	female	EUR	2.763	5.286	6.01E-01	combined
pc5	female	EUR	-8.689	20.119	6.66E-01	combined
pc6	female	EUR	24.988	15.004	9.58E-02	combined
pc7	female	EUR	-8.340	11.323	4.61E-01	combined
pc8	female	EUR	43.078	20.139	3.24E-02	combined
pc9	female	EUR	20.015	21.425	3.50E-01	combined
pc10	female	EUR	22.972	21.516	2.86E-01	combined
pc11	female	EUR	14.562	21.110	4.90E-01	combined
pc12	female	EUR	7.765	21.245	7.15E-01	combined
pc13	female	EUR	-2.228	20.868	9.15E-01	combined
pc14	female	EUR	-12.892	20.449	5.28E-01	combined
pc15	female	EUR	-8.697	20.741	6.75E-01	combined
pc16	female	EUR	-2.769	20.679	8.93E-01	combined
cancer history	all	not EUR	1.771	0.065	1.14E-162	combined
age at diagnosis	all	not EUR	-0.005	0.002	4.27E-02	combined
sex	all	not EUR	0.393	0.062	3.21E-10	combined
annual income	all	not EUR	0.047	0.016	2.77E-03	combined
insurance	all	not EUR	0.520	0.221	1.87E-02	combined
education	all	not EUR	0.076	0.032	1.77E-02	combined
living	all	not EUR	-0.271	0.110	1.38E-02	combined
alcohol daily	all	not EUR	-0.210	0.058	2.76E-04	combined
alcohol yearly	all	not EUR	0.049	0.029	8.82E-02	combined
smoking daily	all	not EUR	0.014	0.003	2.72E-05	combined
smoking yearly	all	not EUR	0.004	0.003	1.33E-01	combined
latitude	all	not EUR	0.016	0.007	2.21E-02	combined
longitude	all	not EUR	0.008	0.002	7.81E-05	combined
hispanic	all	not EUR	12.318	171.342	9.43E-01	combined
african	all	not EUR	12.370	171.343	9.42E-01	combined
european	all	not EUR	13.007	171.342	9.39E-01	combined

admixed	all	not EUR	11.518	171.342	9.46E-01	combined
asian	all	not EUR	10.701	171.342	9.50E-01	combined
middle eastern	all	not EUR	10.450	171.343	9.51E-01	combined
pc1	all	not EUR	5.097	1.024	6.48E-07	combined
pc2	all	not EUR	1.803	1.466	2.19E-01	combined
pc3	all	not EUR	-7.971	1.846	1.57E-05	combined
pc4	all	not EUR	0.401	2.569	8.76E-01	combined
pc5	all	not EUR	2.081	7.284	7.75E-01	combined
pc6	all	not EUR	4.242	7.049	5.47E-01	combined
pc7	all	not EUR	-8.842	6.211	1.55E-01	combined
pc8	all	not EUR	16.523	12.419	1.83E-01	combined
pc9	all	not EUR	-17.602	13.668	1.98E-01	combined
pc10	all	not EUR	-5.974	13.604	6.61E-01	combined
pc11	all	not EUR	7.946	13.461	5.55E-01	combined
pc12	all	not EUR	1.951	13.698	8.87E-01	combined
pc13	all	not EUR	21.014	13.767	1.27E-01	combined
pc14	all	not EUR	-18.693	13.628	1.70E-01	combined
pc15	all	not EUR	19.132	13.601	1.60E-01	combined
pc16	all	not EUR	16.233	13.735	2.37E-01	combined
avanafil	all	not EUR	-12.913	537.292	9.81E-01	combined
sildenafil	all	not EUR	0.497	0.182	6.46E-03	combined
tadalafil	all	not EUR	0.187	0.222	4.00E-01	combined
cancer history	male	not EUR	1.739	0.083	2.41E-97	combined
age at diagnosis	male	not EUR	-0.006	0.003	4.84E-02	combined
annual income	male	not EUR	0.068	0.020	6.33E-04	combined
insurance	male	not EUR	0.237	0.244	3.31E-01	combined
education	male	not EUR	0.155	0.044	4.05E-04	combined
living	male	not EUR	-0.287	0.143	4.45E-02	combined
alcohol daily	male	not EUR	-0.067	0.063	2.91E-01	combined
alcohol yearly	male	not EUR	-0.001	0.034	9.81E-01	combined
smoking daily	male	not EUR	0.019	0.004	6.95E-07	combined
smoking yearly	male	not EUR	0.004	0.003	2.55E-01	combined
latitude	male	not EUR	0.020	0.009	1.86E-02	combined
longitude	male	not EUR	0.006	0.003	2.14E-02	combined
hispanic	male	not EUR	12.735	226.821	9.55E-01	combined
african	male	not EUR	13.016	226.821	9.54E-01	combined
european	male	not EUR	13.263	226.821	9.53E-01	combined
admixed	male	not EUR	11.431	226.821	9.60E-01	combined
asian	male	not EUR	10.951	226.820	9.61E-01	combined

middle eastern	male	not EUR	10.929	226.821	9.62E-01	combined
pc1	male	not EUR	5.966	1.488	6.12E-05	combined
pc2	male	not EUR	2.905	2.054	1.57E-01	combined
pc3	male	not EUR	-8.404	2.541	9.42E-04	combined
pc4	male	not EUR	1.996	3.317	5.47E-01	combined
pc5	male	not EUR	-5.015	9.589	6.01E-01	combined
pc6	male	not EUR	23.951	11.261	3.34E-02	combined
pc7	male	not EUR	-18.511	9.562	5.29E-02	combined
pc8	male	not EUR	28.216	16.414	8.56E-02	combined
pc9	male	not EUR	-6.978	19.062	7.14E-01	combined
pc10	male	not EUR	-13.384	19.217	4.86E-01	combined
pc11	male	not EUR	-26.532	18.880	1.60E-01	combined
pc12	male	not EUR	2.192	19.191	9.09E-01	combined
pc13	male	not EUR	34.445	19.144	7.20E-02	combined
pc14	male	not EUR	-17.623	19.060	3.55E-01	combined
pc15	male	not EUR	6.788	18.953	7.20E-01	combined
pc16	male	not EUR	16.452	19.241	3.93E-01	combined
avanafil	male	not EUR	-12.878	480.140	9.79E-01	combined
sildenafil	male	not EUR	0.542	0.168	1.28E-03	combined
tadalafil	male	not EUR	0.190	0.205	3.54E-01	combined
cancer history	female	not EUR	1.811	0.095	6.35E-81	combined
age at diagnosis	female	not EUR	-0.005	0.003	1.48E-01	combined
annual income	female	not EUR	0.023	0.023	3.24E-01	combined
insurance	female	not EUR	0.941	0.408	2.10E-02	combined
education	female	not EUR	0.011	0.044	8.12E-01	combined
living	female	not EUR	-0.228	0.157	1.48E-01	combined
alcohol daily	female	not EUR	-0.458	0.102	6.91E-06	combined
alcohol yearly	female	not EUR	0.128	0.045	4.52E-03	combined
smoking daily	female	not EUR	0.005	0.006	4.11E-01	combined
smoking yearly	female	not EUR	0.007	0.004	1.06E-01	combined
latitude	female	not EUR	0.013	0.011	2.22E-01	combined
longitude	female	not EUR	0.011	0.003	9.23E-04	combined
hispanic	female	not EUR	11.890	243.144	9.61E-01	combined
african	female	not EUR	11.822	243.144	9.61E-01	combined
european	female	not EUR	12.747	243.144	9.58E-01	combined
admixed	female	not EUR	11.443	243.144	9.62E-01	combined
asian	female	not EUR	10.366	243.143	9.66E-01	combined
middle eastern	female	not EUR	9.834	243.144	9.68E-01	combined
pc1	female	not EUR	4.685	1.327	4.15E-04	combined

pc2	female	not EUR	0.707	1.965	7.19E-01	combined
pc3	female	not EUR	-7.958	2.502	1.47E-03	combined
pc4	female	not EUR	-1.629	3.746	6.64E-01	combined
pc5	female	not EUR	8.445	9.775	3.88E-01	combined
pc6	female	not EUR	-5.733	7.474	4.43E-01	combined
pc7	female	not EUR	-5.612	7.203	4.36E-01	combined
pc8	female	not EUR	7.484	16.822	6.56E-01	combined
pc9	female	not EUR	-27.924	18.260	1.26E-01	combined
pc10	female	not EUR	-0.775	18.052	9.66E-01	combined
pc11	female	not EUR	34.096	18.103	5.96E-02	combined
pc12	female	not EUR	1.806	18.274	9.21E-01	combined
pc13	female	not EUR	11.982	18.549	5.18E-01	combined
pc14	female	not EUR	-21.104	18.238	2.47E-01	combined
pc15	female	not EUR	28.422	18.205	1.18E-01	combined
pc16	female	not EUR	15.161	18.359	4.09E-01	combined

Table S5: Stratification of individuals with skin cancer across all combinations of skin cancer categories, populations, and sexes using a logistic regression

Prediction accuracy for the 45 logistic regression models shown in Figure S3. Combinations of all cancer types, populations and sexes were analyzed. Shown are PPV, NPV, sensitivity, specificity, precision, recall, F1 statistic, area under the ROC curve (ROC AUC), area under the precision-recall curve (AUC PR) and threshold to discriminate between positive and negative predictions. Accuracies were calculated on the five-fold cross-validation training set (see Methods).

Cancer type	Population	Sex	PPV	NPV	Sensitivity	Specificity	Precision	Recall	F1	Threshold	ROC AUC	AUC PR
any	ALL	all	0.097	0.991	0.887	0.596	0.097	0.887	0.175	0.039	0.825	0.898
melanoma	ALL	all	0.027	0.997	0.787	0.731	0.027	0.787	0.051	0.010	0.840	0.975
SCC	ALL	all	0.044	0.996	0.808	0.755	0.044	0.808	0.083	0.014	0.856	0.965
BCC	ALL	all	0.061	0.995	0.867	0.646	0.061	0.867	0.115	0.027	0.828	0.942
other	ALL	all	0.039	0.995	0.791	0.697	0.039	0.791	0.074	0.013	0.829	0.961
any	EUR	all	0.149	0.959	0.625	0.710	0.149	0.625	0.241	0.071	0.724	0.867
melanoma	EUR	all	0.033	0.992	0.641	0.716	0.033	0.641	0.063	0.014	0.728	0.970
SCC	EUR	all	0.052	0.992	0.726	0.709	0.052	0.726	0.096	0.019	0.787	0.953
BCC	EUR	all	0.079	0.981	0.721	0.629	0.079	0.721	0.142	0.038	0.735	0.923
other	EUR	all	0.058	0.990	0.695	0.716	0.058	0.695	0.106	0.022	0.773	0.946
any	not EUR	all	0.082	0.998	0.844	0.882	0.082	0.844	0.149	0.016	0.918	0.959
melanoma	not EUR	all	0.020	0.999	0.848	0.880	0.020	0.848	0.040	0.004	0.904	0.989
SCC	not EUR	all	0.018	0.999	0.827	0.852	0.018	0.827	0.036	0.003	0.880	0.989
BCC	not EUR	all	0.046	0.999	0.838	0.888	0.046	0.838	0.086	0.008	0.930	0.977
other	not EUR	all	0.032	0.999	0.839	0.899	0.032	0.839	0.062	0.007	0.916	0.985
any	ALL	Male	0.117	0.990	0.908	0.575	0.117	0.908	0.208	0.052	0.826	0.875
melanoma	ALL	Male	0.033	0.997	0.833	0.679	0.033	0.833	0.063	0.012	0.837	0.967
SCC	ALL	Male	0.065	0.995	0.840	0.724	0.065	0.840	0.121	0.021	0.855	0.945
BCC	ALL	Male	0.083	0.995	0.903	0.641	0.083	0.903	0.153	0.036	0.843	0.923
other	ALL	Male	0.056	0.994	0.810	0.709	0.056	0.810	0.105	0.019	0.843	0.946
any	EUR	Male	0.164	0.953	0.701	0.629	0.164	0.701	0.266	0.082	0.724	0.839
melanoma	EUR	Male	0.067	0.989	0.513	0.857	0.067	0.513	0.118	0.027	0.754	0.959
SCC	EUR	Male	0.067	0.989	0.792	0.629	0.067	0.792	0.123	0.024	0.785	0.933
BCC	EUR	Male	0.097	0.974	0.754	0.564	0.097	0.754	0.172	0.047	0.706	0.902
other	EUR	Male	0.099	0.983	0.585	0.820	0.099	0.585	0.169	0.042	0.762	0.929
any	not EUR	Male	0.107	0.998	0.929	0.855	0.107	0.929	0.192	0.016	0.937	0.939
melanoma	not EUR	Male	0.017	1.000	0.905	0.816	0.017	0.905	0.033	0.002	0.914	0.989
SCC	not EUR	Male	0.037	1.000	0.949	0.839	0.037	0.949	0.071	0.004	0.936	0.979
BCC	not EUR	Male	0.063	0.999	0.935	0.856	0.063	0.935	0.118	0.007	0.948	0.964
other	not EUR	Male	0.051	0.999	0.788	0.920	0.051	0.788	0.096	0.012	0.901	0.981
any	ALL	Female	0.085	0.987	0.770	0.687	0.085	0.770	0.154	0.043	0.807	0.921
melanoma	ALL	Female	0.020	0.998	0.831	0.684	0.020	0.831	0.039	0.007	0.822	0.981
SCC	ALL	Female	0.027	0.998	0.772	0.767	0.027	0.772	0.052	0.011	0.830	0.980
BCC	ALL	Female	0.049	0.996	0.854	0.665	0.049	0.854	0.092	0.023	0.827	0.956
other	ALL	Female	0.030	0.997	0.798	0.711	0.030	0.798	0.057	0.012	0.837	0.971
any	EUR	Female	0.123	0.965	0.574	0.740	0.123	0.574	0.203	0.063	0.713	0.895
melanoma	EUR	Female	0.037	0.993	0.514	0.842	0.037	0.514	0.069	0.017	0.716	0.976
SCC	EUR	Female	0.027	0.996	0.852	0.567	0.027	0.852	0.053	0.010	0.774	0.972
BCC	EUR	Female	0.051	0.988	0.834	0.478	0.051	0.834	0.097	0.025	0.714	0.942
other	EUR	Female	0.047	0.990	0.609	0.757	0.047	0.609	0.087	0.021	0.746	0.959
any	not EUR	Female	0.032	0.998	0.844	0.793	0.032	0.844	0.062	0.007	0.877	0.975
melanoma	not EUR	Female	0.031	1.000	0.826	0.938	0.031	0.826	0.060	0.006	0.923	0.990
SCC	not EUR	Female	0.009	1.000	0.900	0.795	0.009	0.900	0.018	0.002	0.892	0.994
BCC	not EUR	Female	0.018	1.000	0.905	0.894	0.018	0.905	0.036	0.006	0.940	0.991
other	not EUR	Female	0.018	1.000	1.000	0.792	0.018	1.000	0.036	0.002	0.953	0.984

Table S6: Stratification of individuals with skin cancer across all combinations of skin cancer categories, populations, and sexes using XGBoost.

Prediction accuracy for the 45 prediction XGBoost models shown in Figure 5. Combinations of all cancer types, populations and sexes were analyzed. Shown are PPV, NPV, sensitivity, specificity, precision, recall, F1 statistic, area under the ROC curve (ROC AUC), area under the precision-recall curve (AUC PR) and threshold to discriminate between positive and negative predictions. Accuracies were calculated on the five-fold cross-validated training set (see Methods).

Cancer type	Population	Sex	ROC AUC	AUC PR	PPV	NPV	Sensitivity	Specificity	Precision	Recall	F1	Threshold
any	ALL	all	0.972	0.974	0.809	1.000	0.993	0.988	0.809	0.993	0.892	0.063
melanoma	ALL	all	0.831	0.883	0.112	1.000	0.966	0.929	0.112	0.966	0.201	0.010
SCC	ALL	all	0.939	0.955	0.404	1.000	0.982	0.980	0.404	0.982	0.572	0.019
BCC	ALL	all	0.963	0.965	0.747	1.000	0.983	0.991	0.747	0.983	0.849	0.037
other	ALL	all	0.903	0.956	0.361	1.000	0.974	0.973	0.361	0.974	0.526	0.021
any	EUR	all	0.975	0.981	0.885	0.999	0.993	0.990	0.885	0.993	0.936	0.078
melanoma	EUR	all	0.827	0.896	0.122	0.999	0.953	0.896	0.122	0.953	0.216	0.011
SCC	EUR	all	0.935	0.965	0.514	1.000	0.986	0.980	0.514	0.986	0.676	0.023
BCC	EUR	all	0.958	0.977	0.765	1.000	0.992	0.987	0.765	0.992	0.864	0.040
other	EUR	all	0.906	0.967	0.368	1.000	0.988	0.958	0.368	0.988	0.536	0.022
any	not EUR	all	0.906	0.942	0.103	1.000	0.990	0.892	0.103	0.990	0.186	0.005
melanoma	not EUR	all	0.880	0.772	0.012	1.000	0.935	0.769	0.012	0.935	0.023	0.000
SCC	not EUR	all	0.904	0.830	0.014	1.000	0.981	0.763	0.014	0.981	0.027	0.000
BCC	not EUR	all	0.899	0.885	0.046	1.000	0.980	0.871	0.046	0.980	0.088	0.001
other	not EUR	all	0.903	0.868	0.021	1.000	0.968	0.823	0.021	0.968	0.042	0.000
any	ALL	Male	0.968	0.980	0.834	0.999	0.983	0.988	0.834	0.983	0.902	0.079
melanoma	ALL	Male	0.878	0.910	0.093	1.000	0.978	0.875	0.093	0.978	0.170	0.007
SCC	ALL	Male	0.955	0.990	0.584	1.000	1.000	0.984	0.584	1.000	0.738	0.026
BCC	ALL	Male	0.945	0.968	0.721	0.999	0.986	0.986	0.721	0.986	0.833	0.040
other	ALL	Male	0.888	0.942	0.231	0.999	0.973	0.930	0.231	0.973	0.374	0.014
any	EUR	Male	0.971	0.984	0.855	0.999	0.988	0.983	0.855	0.988	0.917	0.087
melanoma	EUR	Male	0.890	0.929	0.108	1.000	0.981	0.839	0.108	0.981	0.195	0.007
SCC	EUR	Male	0.936	0.966	0.433	1.000	0.996	0.957	0.433	0.996	0.604	0.020
BCC	EUR	Male	0.941	0.962	0.741	0.998	0.970	0.979	0.741	0.970	0.840	0.051
other	EUR	Male	0.913	0.949	0.243	0.999	0.969	0.898	0.243	0.969	0.389	0.014
any	not EUR	Male	0.902	0.912	0.105	1.000	0.982	0.843	0.105	0.982	0.190	0.002
melanoma	not EUR	Male	0.905	0.815	0.021	1.000	0.905	0.851	0.021	0.905	0.040	0.000
SCC	not EUR	Male	0.923	0.905	0.027	1.000	1.000	0.764	0.027	1.000	0.052	0.000
BCC	not EUR	Male	0.927	0.912	0.060	1.000	0.984	0.841	0.060	0.984	0.113	0.000
other	not EUR	Male	0.909	0.861	0.039	1.000	0.939	0.873	0.039	0.939	0.075	0.000
any	ALL	Female	0.955	0.966	0.748	0.999	0.984	0.987	0.748	0.984	0.850	0.045
melanoma	ALL	Female	0.804	0.860	0.039	0.999	0.946	0.821	0.039	0.946	0.076	0.004
SCC	ALL	Female	0.908	0.959	0.203	1.000	0.972	0.968	0.203	0.972	0.336	0.009
BCC	ALL	Female	0.940	0.958	0.603	1.000	0.981	0.987	0.603	0.981	0.747	0.029
other	ALL	Female	0.872	0.927	0.145	0.999	0.945	0.938	0.145	0.945	0.251	0.010
any	EUR	Female	0.967	0.979	0.827	1.000	0.993	0.987	0.827	0.993	0.903	0.059
melanoma	EUR	Female	0.911	0.914	0.055	0.999	0.964	0.805	0.055	0.964	0.104	0.004
SCC	EUR	Female	0.890	0.941	0.363	0.999	0.959	0.976	0.363	0.959	0.527	0.014
BCC	EUR	Female	0.957	0.973	0.828	0.999	0.980	0.993	0.828	0.980	0.897	0.040
other	EUR	Female	0.871	0.912	0.149	0.999	0.953	0.893	0.149	0.953	0.258	0.010
any	not EUR	Female	0.909	0.897	0.035	1.000	0.961	0.786	0.035	0.961	0.068	0.001
melanoma	not EUR	Female	0.935	0.957	0.017	1.000	0.957	0.864	0.017	0.957	0.033	0.000
SCC	not EUR	Female	0.900	0.808	0.011	1.000	1.000	0.809	0.011	1.000	0.022	0.000
BCC	not EUR	Female	0.976	0.953	0.016	1.000	0.952	0.867	0.016	0.952	0.031	0.000
other	not EUR	Female	0.865	0.769	0.015	1.000	0.973	0.754	0.015	0.973	0.030	0.000

Table S7: Evaluation of specific variables on XGBoost multiethnic model by removing one variable at a time from training set

Each prediction in Figure S4 was performed by removing one set of variables, then re-training the XGBoost model and addressing its accuracy on the five-fold cross-validation training set. Variables include: sex, age, cancer history, SDOH, lifestyle, genotype PCs, geo=longitude and latitude, PDE5A inhibitors, and self-reported ancestry. Prediction accuracy is divided by sex (All, Male, Female) and population (All, EUR, not EUR). The All represents all individuals (both sexes and all populations). Shown are PPV, NPV, sensitivity, specificity, precision, recall, F1 statistic, NPV, area under the ROC curve (ROC AUC), area under the precision-recall curve (AUC PR), sensitivity and specificity.

Dataset	PPV	NPV	Sensitivity	Specificity	Precision	Recall	F1	ROC AUC	ROC PR	Threshold
sex: All	0.799	1.000	0.992	0.988	0.799	0.992	0.885	0.999	0.850	0.054
sex: Male	0.827	1.000	0.995	0.987	0.827	0.995	0.904	0.999	0.823	0.054
sex: Female	0.773	1.000	0.989	0.988	0.773	0.989	0.868	0.998	0.869	0.054
sex: EUR	0.813	1.000	0.995	0.982	0.813	0.995	0.895	0.998	0.793	0.054
sex: not EUR	0.703	1.000	0.968	0.995	0.703	0.968	0.814	0.999	0.946	0.054
age: All	0.122	0.997	0.953	0.665	0.122	0.953	0.217	0.917	0.870	0.035
age: Male	0.137	0.997	0.969	0.617	0.137	0.969	0.240	0.919	0.843	0.035
age: Female	0.110	0.996	0.937	0.695	0.110	0.937	0.198	0.914	0.888	0.035
age: EUR	0.118	0.992	0.957	0.434	0.118	0.957	0.211	0.863	0.827	0.035
age: not EUR	0.168	0.999	0.920	0.944	0.168	0.920	0.284	0.963	0.950	0.035
cancer history: All	0.867	1.000	0.994	0.993	0.867	0.994	0.926	0.999	0.850	0.053
cancer history: Male	0.857	1.000	0.995	0.990	0.857	0.995	0.921	0.999	0.823	0.053
cancer history: Female	0.877	1.000	0.993	0.994	0.877	0.993	0.931	0.999	0.869	0.053
cancer history: EUR	0.890	1.000	0.997	0.990	0.890	0.997	0.940	0.998	0.793	0.053
cancer history: not EUR	0.720	1.000	0.973	0.995	0.720	0.973	0.828	0.999	0.946	0.053
SDOH: All	0.819	1.000	0.992	0.989	0.819	0.992	0.897	0.999	0.850	0.060
SDOH: Male	0.818	1.000	0.995	0.986	0.818	0.995	0.898	0.998	0.823	0.060
SDOH: Female	0.819	1.000	0.989	0.991	0.819	0.989	0.896	0.999	0.869	0.060
SDOH: EUR	0.835	1.000	0.995	0.984	0.835	0.995	0.908	0.999	0.793	0.060
SDOH: not EUR	0.709	1.000	0.973	0.995	0.709	0.973	0.821	0.999	0.946	0.060
lifestyle: All	0.801	1.000	0.996	0.988	0.801	0.996	0.888	0.999	0.850	0.051
lifestyle: Male	0.801	1.000	0.996	0.984	0.801	0.996	0.888	0.999	0.823	0.051
lifestyle: Female	0.800	1.000	0.995	0.990	0.800	0.995	0.887	0.999	0.869	0.051
lifestyle: EUR	0.816	1.000	0.998	0.982	0.816	0.998	0.898	0.998	0.793	0.051
lifestyle: not EUR	0.694	1.000	0.979	0.995	0.694	0.979	0.812	0.999	0.946	0.051
genotype: All	0.798	1.000	0.995	0.988	0.798	0.995	0.886	0.999	0.850	0.054
genotype: Male	0.788	1.000	0.996	0.983	0.788	0.996	0.880	0.998	0.823	0.054
genotype: Female	0.808	1.000	0.994	0.990	0.808	0.994	0.891	0.999	0.869	0.054
genotype: EUR	0.821	1.000	0.997	0.983	0.821	0.997	0.900	0.998	0.793	0.054
genotype: not EUR	0.652	1.000	0.979	0.994	0.652	0.979	0.783	0.999	0.946	0.054
geo: All	0.796	1.000	0.994	0.988	0.796	0.994	0.884	0.998	0.850	0.056
geo: Male	0.808	1.000	0.995	0.985	0.808	0.995	0.892	0.998	0.823	0.056
geo: Female	0.785	1.000	0.993	0.989	0.785	0.993	0.877	0.998	0.869	0.056
geo: EUR	0.809	1.000	0.998	0.981	0.809	0.998	0.894	0.998	0.793	0.056
geo: not EUR	0.702	1.000	0.963	0.995	0.702	0.963	0.812	0.998	0.946	0.056
PDE5a inhibitors: All	0.794	1.000	0.994	0.987	0.794	0.994	0.883	0.999	0.850	0.052
PDE5a inhibitors: Male	0.794	1.000	0.996	0.984	0.794	0.996	0.884	0.999	0.823	0.052
PDE5a inhibitors: Female	0.794	1.000	0.992	0.990	0.794	0.992	0.882	0.999	0.869	0.052
PDE5a inhibitors: EUR	0.810	1.000	0.999	0.981	0.810	0.999	0.895	0.998	0.793	0.052
PDE5a inhibitors: not EUR	0.679	0.999	0.957	0.994	0.679	0.957	0.795	0.999	0.946	0.052
self-reported: All	0.808	1.000	0.993	0.988	0.808	0.993	0.891	0.998	0.850	0.069
self-reported: Male	0.817	1.000	0.996	0.986	0.817	0.996	0.898	0.999	0.823	0.069
self-reported: Female	0.800	1.000	0.990	0.990	0.800	0.990	0.885	0.998	0.869	0.069
self-reported: EUR	0.818	1.000	0.999	0.982	0.818	0.999	0.899	0.998	0.793	0.069
self-reported: not EUR	0.737	0.999	0.952	0.996	0.737	0.952	0.831	0.999	0.946	0.069

Table S8: Variable importance in the XGBoost multiethnic model

Metrics assessing the importance of each variable in the XGBoost multiethnic prediction model:

Gain: This metric represents the improvement in accuracy brought by a feature to the model. It measures the average gain in the loss function when the feature is used for splitting a node in the tree. Features with higher gain values are deemed more important as they contribute more to reducing the overall loss.

Cover: Cover refers to the relative quantity of data points associated with a specific feature. It measures the average coverage of instances (samples) associated with the feature when it is used for splitting nodes. Features with higher cover values indicate that they are used more frequently in the decision-making process.

Frequency: Frequency represents the number of times a feature appears in the trees of the ensemble model. It indicates how often a feature is selected for splitting nodes across all trees. Features with higher frequency values are considered more important as they are used more frequently in the decision-making process.

Feature	Gain	Cover	Frequency
age at diagnosis	0.712	0.581	0.354
latitude	0.010	0.032	0.052
longitude	0.012	0.035	0.044
cancer history	0.094	0.077	0.032
pc11	0.003	0.004	0.031
pc2	0.059	0.081	0.030
smoking yearly	0.007	0.011	0.030
pc4	0.003	0.006	0.030
pc15	0.003	0.004	0.028
pc14	0.003	0.003	0.027
pc10	0.003	0.003	0.027
pc12	0.002	0.002	0.027
annual income	0.008	0.023	0.026
pc5	0.003	0.003	0.025
pc3	0.002	0.005	0.025
pc9	0.002	0.003	0.023
pc16	0.002	0.004	0.023
pc1	0.003	0.008	0.023
pc6	0.003	0.008	0.021
pc13	0.002	0.003	0.021
pc8	0.002	0.004	0.019
pc7	0.003	0.003	0.017
education	0.008	0.007	0.016
smoking daily	0.007	0.006	0.014

alcohol yearly	0.003	0.008	0.009
sex	0.008	0.013	0.009
alcohol daily	0.002	0.008	0.006
living	0.004	0.013	0.004
european	0.023	0.029	0.003
sildenafil	0.001	0.005	0.003
tadalafil	0.000	0.003	0.003
insurance	0.001	0.004	0.001
hispanic	0.000	0.000	0.000

Table S9: Characterization of true positive and false positive individuals in the validation set

We tested each of the variables (features) using a logistic regression model for differences between true positives and false positives. Shown are the effect sizes, their standard errors, two-sided p-values (*glm* function in R) and p-values adjusted with Bonferroni's method. The positive effect size for age means the true positives were older and the negative effect size means the false positives were less likely to have a history of cancer.

Feature	Effect size	Standard error	p-value	Adjusted p-value (Bonferroni)
(Intercept)	1.332	1.514	3.79E-01	1.00E+00
age	0.045	0.005	1.09E-16	3.69E-15
cancer history	-1.500	0.134	6.07E-29	2.06E-27
pc2	1.194	5.117	8.16E-01	1.00E+00
european	-0.462	0.934	6.21E-01	1.00E+00
lng	0.001	0.005	8.41E-01	1.00E+00
lat	-0.042	0.017	1.56E-02	5.32E-01
education	0.074	0.067	2.72E-01	1.00E+00
sex	-0.336	0.133	1.13E-02	3.84E-01
annual income	0.011	0.035	7.54E-01	1.00E+00
smoking yearly	-0.007	0.006	2.54E-01	1.00E+00
smoking daily	0.008	0.008	3.20E-01	1.00E+00
living	-0.311	0.252	2.17E-01	1.00E+00
pc1	7.885	2.775	4.48E-03	1.52E-01
pc4	1.263	9.062	8.89E-01	1.00E+00
pc7	-26.944	27.149	3.21E-01	1.00E+00
alcohol yearly	0.228	0.058	7.35E-05	2.50E-03
pc11	7.371	55.475	8.94E-01	1.00E+00

pc14	-85.589	55.438	1.23E-01	1.00E+00
pc6	57.378	33.118	8.32E-02	1.00E+00
pc15	90.122	55.735	1.06E-01	1.00E+00
pc10	36.576	51.850	4.81E-01	1.00E+00
pc5	45.725	23.801	5.47E-02	1.00E+00
alcohol daily	-0.378	0.117	1.20E-03	4.08E-02
pc12	17.191	56.049	7.59E-01	1.00E+00
pc3	-11.194	6.629	9.13E-02	1.00E+00
pc9	53.667	53.598	3.17E-01	1.00E+00
pc16	52.758	55.373	3.41E-01	1.00E+00
pc8	86.488	49.855	8.28E-02	1.00E+00
pc13	17.382	51.215	7.34E-01	1.00E+00
sildenafil	1.074	0.515	3.71E-02	1.00E+00
insurance	-1.443	0.920	1.17E-01	1.00E+00
tadalafil	0.214	0.543	6.94E-01	1.00E+00
hispanic	0.468	1.031	6.50E-01	1.00E+00

Table S10: Stratification of individuals with skin cancer who have missing information

Prediction accuracy of the XGBoost multiethnic model on individuals with missing information. The data sets are divided by: All (all individuals), sex (male, female) and population (EUR, non-EUR). Shown are PPV, NPV, sensitivity, specificity, precision, recall, F1 statistic, and threshold. The last column describes whether the prediction was performed on: 1) all AoU individuals with missing information (set = “missing”, Figure 5C); or 2) all individuals in the validation set, having each set of variables artificially set to “NA” (set = “validation”, Figure 5D).

Dataset	PPV	NPV	Sensitivity	Specificity	Precision	Recall	F1	Threshold	Set
Missing SDOH: All	0.809	0.998	0.946	0.992	0.809	0.946	0.872	0.063	missing
Missing SDOH: Male	0.783	0.998	0.960	0.990	0.783	0.960	0.863	0.063	missing
Missing SDOH: Female	0.832	0.998	0.934	0.994	0.832	0.934	0.880	0.063	missing
Missing SDOH: EUR	0.859	0.998	0.976	0.985	0.859	0.976	0.914	0.063	missing
Missing SDOH: not EUR	0.652	0.998	0.838	0.995	0.652	0.838	0.733	0.063	missing
sex: All	0.860	1.000	0.993	0.992	0.860	0.993	0.921	0.081	validation
sex: Male	0.864	1.000	0.995	0.990	0.864	0.995	0.925	0.081	validation
sex: Female	0.855	1.000	0.990	0.993	0.855	0.990	0.918	0.081	validation
sex: EUR	0.878	1.000	0.997	0.989	0.878	0.997	0.934	0.081	validation
sex: not EUR	0.738	0.999	0.957	0.996	0.738	0.957	0.833	0.081	validation
age: All	0.865	1.000	0.993	0.992	0.865	0.993	0.924	0.093	validation
age: Male	0.848	1.000	0.996	0.989	0.848	0.996	0.916	0.093	validation
age: Female	0.881	1.000	0.989	0.995	0.881	0.989	0.932	0.093	validation
age: EUR	0.880	1.000	0.997	0.989	0.880	0.997	0.935	0.093	validation
age: not EUR	0.759	0.999	0.957	0.996	0.759	0.957	0.847	0.093	validation
cancer history: All	0.866	0.999	0.989	0.993	0.866	0.989	0.924	0.044	validation
cancer history: Male	0.859	0.999	0.989	0.990	0.859	0.989	0.919	0.044	validation
cancer history: Female	0.873	1.000	0.989	0.994	0.873	0.989	0.928	0.044	validation
cancer history: EUR	0.883	1.000	0.997	0.990	0.883	0.997	0.937	0.044	validation
cancer history: not EUR	0.744	0.999	0.926	0.996	0.744	0.926	0.825	0.044	validation
SDOH: All	0.860	0.999	0.983	0.992	0.860	0.983	0.917	0.036	validation
SDOH: Male	0.850	0.999	0.991	0.989	0.850	0.991	0.915	0.036	validation
SDOH: Female	0.870	0.999	0.975	0.994	0.870	0.975	0.919	0.036	validation
SDOH: EUR	0.884	0.999	0.986	0.990	0.884	0.986	0.932	0.036	validation
SDOH: not EUR	0.706	0.999	0.957	0.995	0.706	0.957	0.813	0.036	validation
lifestyle: All	0.877	1.000	0.990	0.993	0.877	0.990	0.930	0.101	validation
lifestyle: Male	0.858	1.000	0.995	0.990	0.858	0.995	0.921	0.101	validation
lifestyle: Female	0.896	0.999	0.986	0.995	0.896	0.986	0.939	0.101	validation
lifestyle: EUR	0.893	1.000	0.996	0.991	0.893	0.996	0.942	0.101	validation

lifestyle: not EUR	0.764	0.999	0.947	0.996	0.764	0.947	0.846	0.101	validation
genotype: All	0.456	0.998	0.964	0.944	0.456	0.964	0.619	0.006	validation
genotype: Male	0.466	0.999	0.980	0.930	0.466	0.980	0.631	0.006	validation
genotype: Female	0.447	0.998	0.949	0.953	0.447	0.949	0.608	0.006	validation
genotype: EUR	0.543	0.997	0.966	0.935	0.543	0.966	0.695	0.006	validation
genotype: not EUR	0.201	0.999	0.952	0.954	0.201	0.952	0.332	0.006	validation
geo: All	0.786	0.999	0.986	0.987	0.786	0.986	0.875	0.027	validation
geo: Male	0.766	1.000	0.994	0.981	0.766	0.994	0.865	0.027	validation
geo: Female	0.807	0.999	0.977	0.991	0.807	0.977	0.884	0.027	validation
geo: EUR	0.811	0.999	0.988	0.982	0.811	0.988	0.891	0.027	validation
geo: not EUR	0.631	1.000	0.963	0.993	0.631	0.963	0.762	0.027	validation
PDE5a inhibitors: All	0.865	1.000	0.993	0.992	0.865	0.993	0.924	0.093	validation
PDE5a inhibitors: Male	0.848	1.000	0.996	0.989	0.848	0.996	0.916	0.093	validation
PDE5a inhibitors: Female	0.881	1.000	0.989	0.995	0.881	0.989	0.932	0.093	validation
PDE5a inhibitors: EUR	0.880	1.000	0.997	0.989	0.880	0.997	0.935	0.093	validation
PDE5a inhibitors: not EUR	0.759	0.999	0.957	0.996	0.759	0.957	0.847	0.093	validation
self-reported: All	0.865	1.000	0.993	0.992	0.865	0.993	0.924	0.093	validation
self-reported: Male	0.848	1.000	0.996	0.989	0.848	0.996	0.916	0.093	validation
self-reported: Female	0.881	1.000	0.989	0.995	0.881	0.989	0.932	0.093	validation
self-reported: EUR	0.880	1.000	0.997	0.989	0.880	0.997	0.935	0.093	validation
self-reported: not EUR	0.759	0.999	0.957	0.996	0.759	0.957	0.847	0.093	validation
