# Supplementary material for: A highly accurate risk factor-based XGBoost multiethnic model for identifying patients with skin cancer
Source: Nat Commun. 2025 Oct 29;16:9542. doi: 10.1038/s41467-025-64556-y (PMC12572141; doi:10.1038/s41467-025-64556-y)
Supplement: Supplementary file 3 — Reporting Summary [file 41467_2025_64556_MOESM3_ESM.pdf]

nature portfolio

Corresponding author(s): Matteo D'Antonio, Kelly A. Frazer

Last updated by author(s): Jul 20, 2025

Reporting Summary

Nature Portfolio wishes to improve the reproducibility of the work that we publish. This form provides structure for consistency and transparency in reporting. For further information on Nature Portfolio policies, see our Editorial Policies and the Editorial Policy Checklist.

Statistics

For all statistical analyses, confirm that the following items are present in the figure legend, table legend, main text, or Methods section.

n/a	Confirmed	
☐	☒	The exact sample size (*n*) for each experimental group/condition, given as a discrete number and unit of measurement
☒	☐	A statement on whether measurements were taken from distinct samples or whether the same sample was measured repeatedly
☐	☒	The statistical test(s) used AND whether they are one- or two-sided<br>*Only common tests should be described solely by name; describe more complex techniques in the Methods section.*
☐	☒	A description of all covariates tested
☐	☒	A description of any assumptions or corrections, such as tests of normality and adjustment for multiple comparisons
☐	☒	A full description of the statistical parameters including central tendency (e.g. means) or other basic estimates (e.g. regression coefficient) AND variation (e.g. standard deviation) or associated estimates of uncertainty (e.g. confidence intervals)
☐	☒	For null hypothesis testing, the test statistic (e.g. *F*, *t*, *r*) with confidence intervals, effect sizes, degrees of freedom and *P* value noted<br>*Give P values as exact values whenever suitable.*
☒	☐	For Bayesian analysis, information on the choice of priors and Markov chain Monte Carlo settings
☒	☐	For hierarchical and complex designs, identification of the appropriate level for tests and full reporting of outcomes
☒	☐	Estimates of effect sizes (e.g. Cohen's *d*, Pearson's *r*), indicating how they were calculated

Our web collection on statistics for biologists contains articles on many of the points above.

Software and code

Policy information about availability of computer code

Data collection	All analyses were performed using the resources in the All Of Us Researcher Workbench (https://www.researchallofus.org/data-tools/workbench/). Jupyter notebooks and R 4.4.0 were used. Data was obtained using mysql queries in the bigquery package.
Data analysis	All analyses were performed using the resources in the All Of Us Researcher Workbench (https://www.researchallofus.org/data-tools/workbench/). Jupyter notebooks and R 4.4.0 were used. Analyses were performed in R.<br>The following R packages have been used: bigrquery 1.5.1, tidyverse 2.0.0, plyr 1.8.9, dplyr 1.1.4, dbplyr 2.5.0, data.table 1.15.2, glmnet 4.1.8, SHAPforxgboost 0.1.3, openxlsx 4.2.5.2, caret 6.0-94, xgboost 1.7.7.1.<br><br>Notebooks are available at https://figshare.com/s/6c041849df0ef57eadad.

For manuscripts utilizing custom algorithms or software that are central to the research but not yet described in published literature, software must be made available to editors and reviewers. We strongly encourage code deposition in a community repository (e.g. GitHub). See the Nature Portfolio guidelines for submitting code & software for further information.

Data

Policy information about availability of data

All manuscripts must include a data availability statement. This statement should provide the following information, where applicable:

- Accession codes, unique identifiers, or web links for publicly available datasets
- A description of any restrictions on data availability
- For clinical datasets or third party data, please ensure that the statement adheres to our policy

All analyses were performed on the All of Us Researcher Workbench (https://www.researchallofus.org/data-tools/workbench/) in the "Impact of global and local ancestry on genome-wide association V7 studies" workspace (Controlled Tier data). As per AoU policies, we cannot share participant-level information or data on sets that include <20 participants. All other data is available as Supplemental Tables.

Research involving human participants, their data, or biological material

Policy information about studies with human participants or human data. See also policy information about sex, gender (identity/presentation), and sexual orientation and race, ethnicity and racism.

Reporting on sex and gender	Self-reported sex was used as covariate
Reporting on race, ethnicity, or other socially relevant groupings	Genetic ancestry (calculated from genotype principal components) and self-reported ancestry were used.
Population characteristics	Survey data, drug exposures and condition information (ICD9 and ICD10 codes for skin cancer) were obtained from All Of Us. Further details are provided in the Methods section.
Recruitment	All Of Us participant data was used.
Ethics oversight	We did not generate any new data, but used available information in All Of Us.

Note that full information on the approval of the study protocol must also be provided in the manuscript.

Field-specific reporting

Please select the one below that is the best fit for your research. If you are not sure, read the appropriate sections before making your selection.

☒ Life sciences ☐ Behavioural & social sciences ☐ Ecological, evolutionary & environmental sciences

For a reference copy of the document with all sections, see nature.com/documents/nr-reporting-summary-flat.pdf

Life sciences study design

All studies must disclose on these points even when the disclosure is negative.

Sample size	~400,000 All Of Us participants were included: all participants that have genetic data were used for the analysis.
Data exclusions	No data was excluded
Replication	Participants were randomly divided into 70% training, 10% testing and 20% validation sets
Randomization	Division into training, testing and validation was performed randomly.
Blinding	Investigators used the validation set only after the models were trained and tested. Investigators were blind to the division into training, testing and validation

Reporting for specific materials, systems and methods

We require information from authors about some types of materials, experimental systems and methods used in many studies. Here, indicate whether each material, system or method listed is relevant to your study. If you are not sure if a list item applies to your research, read the appropriate section before selecting a response.

Materials & experimental systems

n/a	Involved in the study
☒	☐ Antibodies
☒	☐ Eukaryotic cell lines
☒	☐ Palaeontology and archaeology
☒	☐ Animals and other organisms
☒	☐ Clinical data
☒	☐ Dual use research of concern
☒	☐ Plants

Methods

n/a	Involved in the study
☒	☐ ChIP-seq
☒	☐ Flow cytometry
☒	☐ MRI-based neuroimaging

Plants

Seed stocks	*Report on the source of all seed stocks or other plant material used. If applicable, state the seed stock centre and catalogue number. If plant specimens were collected from the field, describe the collection location, date and sampling procedures.*
Novel plant genotypes	*Describe the methods by which all novel plant genotypes were produced. This includes those generated by transgenic approaches, gene editing, chemical/radiation-based mutagenesis and hybridization. For transgenic lines, describe the transformation method, the number of independent lines analyzed and the generation upon which experiments were performed. For gene-edited lines, describe the editor used, the endogenous sequence targeted for editing, the targeting guide RNA sequence (if applicable) and how the editor was applied.*
Authentication	*Describe any authentication procedures for each seed stock used or novel genotype generated. Describe any experiments used to assess the effect of a mutation and, where applicable, how potential secondary effects (e.g. second site T-DNA insertions, mosiacism, off-target gene editing) were examined.*
